# Engaging Parents in Healthcare Interventions to Improve Paediatric Patient Safety: A Scoping Review

**DOI:** 10.1111/hex.70917

**Published:** 2026-10-12

**Authors:** Thomas Purchase, Nicholas Hodson, Samuel Li, Isobel Joy McFadzean, Joanne Hughes, Philippa Rees, Fiona Wood, Sarah Yardley, Adrian Edwards, Andrew Carson‐Stevens

**Affiliations:** ^1^ Wales Centre for Primary and Emergency Care Research (PRIME Centre Wales), Department of Population Medicine Cardiff University Cardiff UK; ^2^ Mother's Instinct Cambridge UK; ^3^ Population Policy and Practice, UCL, Great Ormond Street Institute of Child Health London UK; ^4^ Department of Population Medicine Cardiff University Cardiff UK; ^5^ Marie Curie Palliative Care Research Department University College London London UK

## Abstract

**Background:**

Paediatric patient safety incidents remain a significant global concern, with parents often playing a crucial role in detecting and preventing harm. Despite international calls for family partnerships in healthcare, evidence on how to meaningfully engage parents as participants in improvement interventions or within their development is limited.

**Objective:**

To summarise and describe healthcare interventions that engage parents to improve key paediatric patient safety issues (relating to medication, communication, diagnosis and assessment, documentation, and administrative processes) and to explore the nature of parental engagement within the studies, including as research participants within the interventions themselves and/or the design and conduct of the study.

**Methods:**

In this scoping review five databases (Medline, EMBASE, CINAHL, Web of Science and Scopus) and grey literature were searched for studies published from 2000 to August 2025. Eligible studies (published in English) described interventions aimed at mitigating key patient safety issues and engaged parents within the intervention and/or the wider study design. Data were independently charted and synthesised narratively using the Patient and Family Engagement (PFE) framework and the International Association for Public Participation (IAP2) Spectrum.

**Results:**

111 studies were included, predominantly from secondary care settings (*n* = 89, 80%), conducting experimental study designs (*n* = 43, 39%) or quality improvement projects (*n* = 36, 32%). Communication and medication safety were the most frequently targeted areas (*n* = 72, 65% and *n* = 55, 50%, respectively). Interventions were categorised into 17 groups, totalling 179 interventions, most commonly involving process standardisation (*n* = 26/111, 23%) or parent‐targeted education (*n* = 25/111, 23%). Outcome measures largely reported parent‐specific factors, such as their perception (*n* = 38/111, 10%) or knowledge (*n* = 21/111, 19%), with minimal evaluation of real‐world safety outcomes. Parental engagement within healthcare interventions was focused on Direct Care (*n* = 108, 97.3%) and most commonly occurred through consultation‐based approaches (*n* = 78, 70.3%). Parental engagement within research activities was primarily classified as Inform (*n* = 66, 59.5%), suggesting limited involvement in research design, development, or delivery.

**Conclusion:**

Parental engagement is essential to improving paediatric patient safety, yet it remains limited, inconsistent, and often tokenistic. Greater partnership with parents is needed, both as contributors to research and as partners in the design and delivery of healthcare services and safety interventions. Embedding engagement within research and quality improvement, alongside standardised approaches to its evaluation and reporting, will be critical to achieving a more inclusive, human‐centred approach in which parental perspectives meaningfully shape safer paediatric care.

**Patient and public contribution:**

The parent patient and public involvement and engagement (PPPIE) contributor for this study met with the principal investigator on several occasions to review the findings and shape the interpretation of the results. They contributed to reviewing and editing the manuscript to improve clarity and accessibility.

## 1 Introduction

Unintended and harmful outcomes arising from healthcare delivery are a frequent international public health issue [1, 2, 3]. This is particularly true amongst children, where 15%–35% of hospitalised children experience a patient safety incident resulting in harm [4], with adverse events occurring across multiple healthcare settings [5].

Engaging parents in the development of patient safety interventions is crucial for minimising risk and preventing healthcare‐associated harm [6]. Implementing interventions with patient and family engagement in care processes can significantly reduce patient safety incidents across care settings [7], but within primary care, for example, there are few interventions successfully engaging families in this way relating to safety [8]. Healthcare providers should recognise the value of parental insights and identify where parents can support the development and delivery of paediatric safety improvements and care quality, especially given international calls for greater family partnership in healthcare [9], including the co‐development of safety interventions [8, 9, 10, 11]. Developing strategies to enable meaningful parental engagement should be a key priority for healthcare organisations [11]. Further, where research may influence parents' and their children's lives, parents want to add their voice to this process, which could provide an important source of empowerment [12]. However, current practice and culture within healthcare systems do not always support parental partnerships or incorporate the family experience [13, 14].

Children (anyone aged under 18 years old) [15], particularly younger children, have a high dependency on parents, guardians, or informal caregivers [16] (henceforth referred to as ‘parents’) to support their healthcare needs. Parents may act as the child's principal advocate and play a vital role in the detection and prevention of unsafe healthcare [17, 18, 19]. This protective role positions parents to strengthen the resilience of healthcare systems, for example, their ability to anticipate and adapt to the potential for surprise and failure [20], and thereby minimise healthcare‐associated harms [21]. Actions could include, for example, identifying unsafe healthcare that might otherwise go undetected [22, 23, 24] and providing relevant information and additional scrutiny to prevent medication‐associated incidents [25]. Mills et al. recommended a proactive assessment of parental concerns as a standard component of clinical care, after identifying that documented parental concerns regarding deterioration had a stronger association with both intensive care unit admission and mechanical ventilation than any documented abnormal vital signs [26]. Therefore, not only engaging parents within the study design process but as research participants (e.g., relating to the intervention) may contribute towards greater system resilience and safer care delivery.

In addition to the recognised value of engaging patients and families in both the processes of healthcare delivery, as well as the development of interventions seeking to improve healthcare, there is a strong moral argument and, some argue, ethical obligation to do so [27]. This perspective aligns with consensus recommendations in paediatrics, which state that decision‐making should be collaborative between parents and clinicians to promote the child's health interests, where parents are presumed to have discretion over making healthcare decisions for their child [28].

A recent study of paediatric safety incident reports in general practice highlighted that parents frequently took action to mitigate safety incidents and prevent harms [29]. Parents had an influence on safety where incidents related to medications, diagnosis and assessment, administrative processes, documentation, and communication, all of which are considered key areas for improvement in the field of patient safety, both within the UK and globally [30, 31, 32, 33, 34, 35, 36, 37, 38, 39]. These areas additionally correlate with where safety incidents and preventable harm occur more broadly within healthcare [2, 3].

To support future research and healthcare organisations in developing and implementing safety interventions that meaningfully engage parents, both within the research process and as research participants within the interventions, this review aims to summarise and describe existing healthcare improvement interventions, initiatives and quality improvement projects. Specifically, it focuses on studies with any form of parental engagement that seek to address or mitigate the key safety areas identified in the aforementioned incident report data analysis [29]. Namely:

- Diagnosis and assessment incidents, for example, relating to delayed diagnoses or an incomplete medical assessment.
- Communication incidents, for example, between parents and healthcare professionals.
- Medication incidents, for example, relating to prescribing or dispensing.
- Documentation incidents, for example, relating to incorrect documentation.
- Administrative incidents, for example, relating to managing appointments, accessing healthcare professionals, or transferring patient information.

As part of this, the review seeks to explore how and where parents were engaged across the studies. This included examining whether parents contributed to the design and development of the interventions and research activities and/or as research participants relating to the interventions themselves.

The parent‐specific role within paediatric safety interventions and their development has not previously been systematically explored within the literature.

Exploring the extent and nature of parental engagement across these domains will provide insight into how parents contribute to intervention development and healthcare practice relating to paedatric safety, while highlighting gaps and priorities for improvement.

## 2 Methods

This scoping review was conducted following the principles described in the Joanna Briggs Institute (JBI) Manual for Evidence Synthesis and is reported in accordance with the Preferred Reporting Items for Systematic Reviews and Meta‐Analyses extension for Scoping Reviews (PRISMA‐ScR) [40, 41].

### 2.1 Inclusion Criteria

#### 2.1.1 Participants

This review considered studies that included parents/guardians/caregivers/family members with parental responsibility for a child aged less than 18 years old.

#### 2.1.2 Concept

Studies described a healthcare intervention, initiative or quality improvement project attempting to improve or mitigate patient safety issues relating to medications, diagnosis and assessment, administrative processes, documentation and/or communication, and described parental engagement relating to the study development or design and/or being a research participant relating to the intervention of interest.

#### 2.1.3 Context

Studies were included from the year 2000 to August 2025 (identifying interventions after the landmark publication To Err is Human [42], and ensuring the interventions were not outdated), in the English language, from any country, where the care setting was either within the community (e.g., primary care) or in hospital settings (e.g., secondary or tertiary care).

#### 2.1.4 Types of Sources

All study designs, relevant grey literature and abstracts without subsequent full‐text publications were included. Studies that did not report original research (e.g., systematic reviews, scoping reviews) were excluded.

Scoping reviews are a descriptive method to provide an overview of the evidence [43], and the purpose of this review was to identify improvement interventions regardless of the quality of evidence supporting them. Studies were not excluded based on methodological quality.

#### 2.1.5 Search Strategy

A search strategy consisting of keywords, phrases, and MeSH terms was initially designed on Ovid Medline (Supporting Information: File 1).

The search strategy was designed using terms relating to the priority safety areas identified in a previous incident report analysis [29], to ensure the findings focused on safety areas where parents can influence care. Additional search terms related to children, intervention development and implementation, and parent engagement. The search strategy was developed with two experienced subject librarians and experts in the field of patient safety, to ensure the terms used were relevant and comprehensive.

A review of the titles, abstracts, key words, and index terms in the articles retrieved from an initial search was undertaken on Ovid Medline. Additional relevant search terms and key words were identified using the InterTASC Information Specialists' Sub‐Group (ISSG) Search Filter Resource [44] and through hand searches of articles in relevant databases and journals (Supporting Information: File 2). All new search terms and key words were incorporated into the search strategy and replicated across four additional databases: EMBASE, CINAHL, Web of Science and Scopus (Supporting Information: File 1).

A grey literature search was conducted [45] using the search terms from the key safety areas. A hand search of Google Scholar and key websites, including the World Health Organization (WHO), the King's Fund, and relevant charities was performed. Our protocol [46] stated that an adapted search strategy would to be applied to Overton, a searchable index of policy documents and guidelines [47]. However, Overton's functionality did not support our search strategy and therefore was not used.

Where abstracts suitable for inclusion had no identifiable subsequent published data, the corresponding author was contacted to establish if publications or presentations had been made. No additional full‐text articles were identified as a result, but the abstracts were included for analysis.

The search was performed in July 2024 and updated in August 2025.

### 2.2 Evidence Screening and Selection

All studies identified were imported to Endnote 21, and duplicates removed. The remaining studies were imported to Rayyan (systematic literature review management software) for screening of titles and abstracts. Screening was undertaken by two independent reviewers (TP and SL) who were blinded to each other's screening decisions. Disagreements between the reviewers were resolved through discussion, and no mediation through an arbiter was required.

All articles included for full text eligibility screening were independently reviewed by two reviewers (TP and NH), who were also blinded. To assess concordance between reviewers, Cohen's kappa inter‐rater reliability statistics were calculated based on the application of the inclusion criteria for full text articles from the database searches. A Cohen's kappa statistic of > 0.6, which indicates substantial agreement, was sought [48]. Full text articles for inclusion were then imported to Microsoft Excel for charting.

The reference lists of all included full text articles and relevant systematic or scoping reviews were reviewed and screened by title and abstract [40].

### 2.3 Data Extraction

Data from eligible full texts papers were independently charted by two reviewers (TP and NH). The variables charted included author(s), year of publication, title, study design, abstract/full text, country, age of children, type of caregiver, care setting, safety area addressed, the type of intervention, intervention details, outcome variables, reported effectiveness, a free‐text description of the parental role within the study, the parent level and continuum of engagement within the intervention and healthcare setting (PFE framework [see below for a description of the framework and what data were extracted]) [49], parent engagement within the research process (IAP2 spectrum [see below for a description of the spectrum and what data were extracted]) [50] and key findings related to our objectives. Where multiple outcomes were measured, e.g., within several cycles of a quality improvement project, the primary outcome and outcomes related to parents were charted.

Pilot data extraction was undertaken by two reviewers on a convenience sample of ten included articles. The results were reviewed, and no major amendments were required to the charting form. The full list of charted results was compared between the two reviewers, and any differences were discussed and mediated, until consensus was reached on each variable and category.

### 2.4 Data Analysis

Due to high levels of heterogeneity (e.g., the methodological and clinical diversity between studies), data were synthesised narratively. To guide the synthesis, the Patient and Family Engagement (PFE) [49] framework and International Association for Public Participation (IAP2) Spectrum [50] were used to explore, describe, and interpret the studies in more detail.

#### 2.4.1 PFE Framework

We used the PFE framework, developed by Carman et al. [49] that was purposefully created to “help inform the development of interventions and policies that support patient and family engagement”. The framework presents the forms parent engagement can take (Figure 1) and has been successfully applied to systematic and scoping reviews exploring patient safety concerns [7, 51, 52]. It defines ‘patient and family engagement’ as “patients, families, their representatives, and health professionals working in active partnership at various levels across the health care system to improve health and health care” [49]. For consistency and simplicity, given its international use and as a developed concept within the PFE framework, the term ‘parent engagement’ is therefore used throughout the remainder of this review.

**Figure 1:**
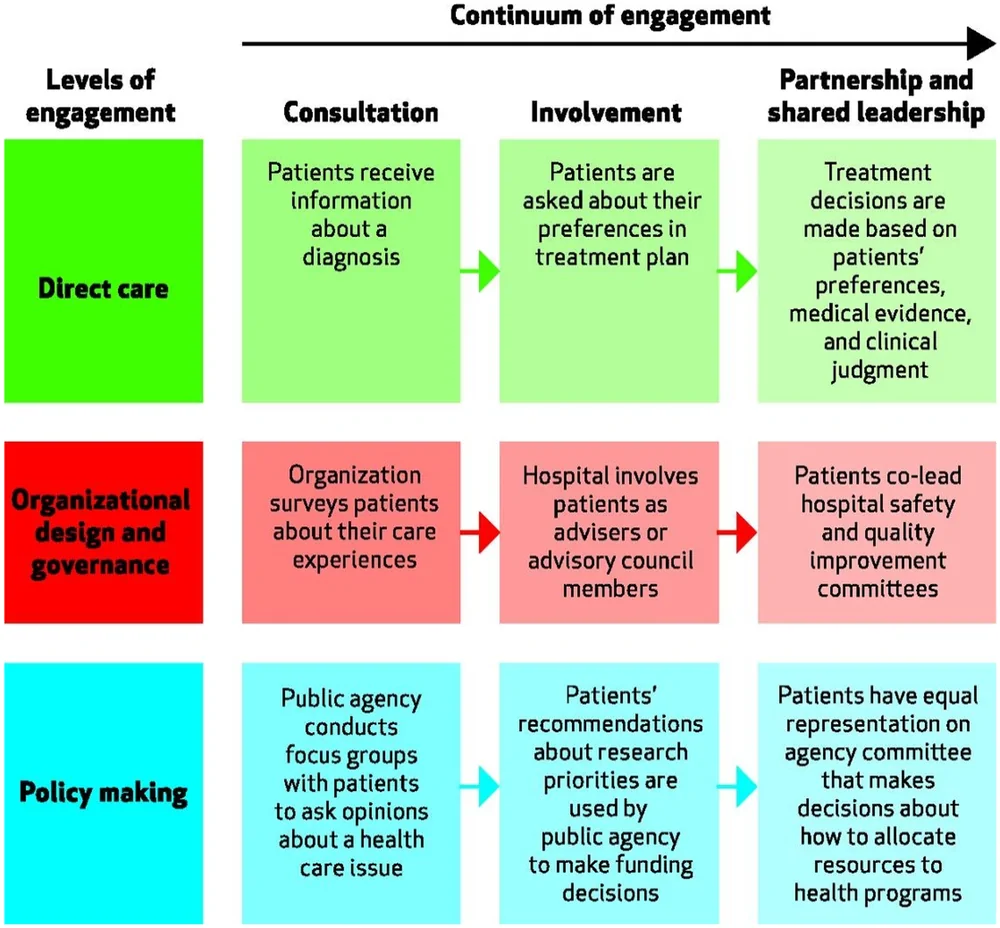
Patient and family engagement framework in health and healthcare. The original framework additionally contains ‘Factors influencing engagement’, but this domain fell outside the scope of this review and has therefore been removed.

The framework conceptualises engagement as a matrix consisting of three levels of engagement and a continuum of engagement (Figure 1). The level of engagement describes where engagement occurs within the healthcare system and identifies the context in which parents are engaged, namely direct care, organisational design and governance, or policy making.

At the Direct Care level, engagement relates to the care of an individual patient and includes activities such as managing health conditions, navigating healthcare services, and interactions between parents and healthcare professionals regarding their child's care. At the Organisational Design and Governance level, engagement involves participation in the design, delivery, evaluation, or improvement of healthcare services, such as membership of advisory councils or quality improvement initiatives. At the Policy‐Making level, engagement involves collaboration with healthcare organisations, funders, researchers, or policy makers to influence service priorities, resource allocation, programmes, or policy decisions.

For each level of engagement, the continuum describes the extent to which parents are engaged, ranging from consultation, through involvement, to partnership and shared leadership, reflecting the direction of information flow and the degree of parental influence in decision‐making processes.

Consultation reflects largely unidirectional information exchange, in which parents receive information and may ask questions or express concerns, but have limited influence on decision‐making. Examples may include receiving education or being present during a ward round. Involvement reflects greater bidirectional communication, where parents' experiences and perspectives are actively sought and considered, giving them some influence over discussions relating to their child's care. Examples may include meaningful participation in family‐centred rounds or communication tools that facilitate parent input. Partnership and Shared Leadership represents the highest level of engagement, which is characterised by shared power, shared responsibility, and collaborative decision‐making between parents and healthcare professionals.

For each included study, the level and continuum of engagement were independently coded based on the role parents played within the intervention, as described by the authors, and the extent to which parental perspectives influenced care or decision‐making processes.

#### 2.4.2 IAP2 Spectrum

To classify the nature and extent of parent engagement within the research activities of each included study, we used the IAP2 Spectrum (Figure 2), a widely recognised framework for characterising public participation within decision‐making and engagement processes [50].

**Figure 2:**
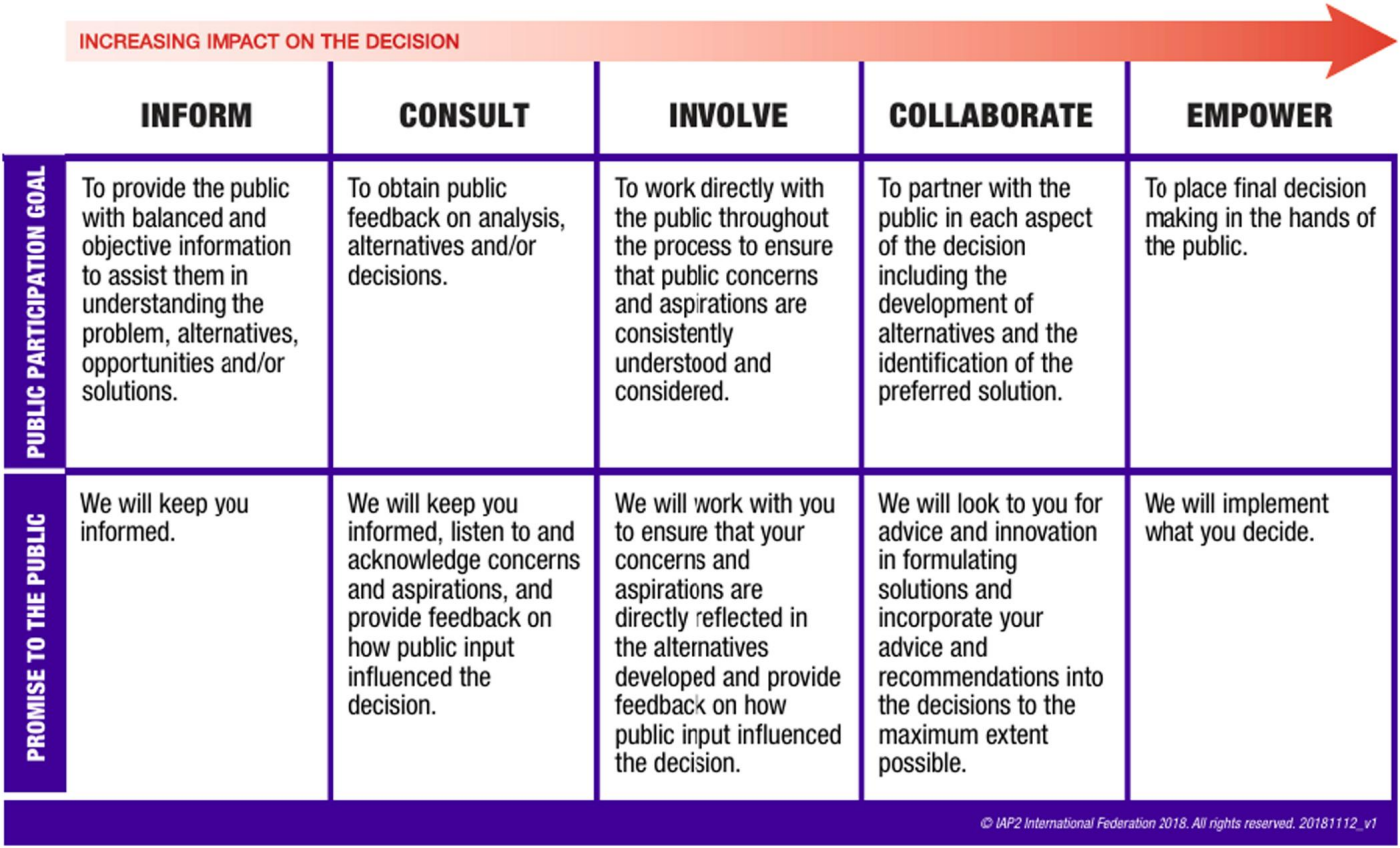
International association for public participation (IAP2) Spectrum. © Federation of International Association for Public Participation 2024. All rights reserved. This work was created with contributions from Lewis Michaelson, Martha Rozelle, and Doug Sarno. www.iap2.org.

In health services research, the IAP2 Spectrum has increasingly been applied to describe the degree of patient, family, and public involvement in research activities, including paediatric health research [53, 54]. The framework provides a structured approach for distinguishing between more consultative and more collaborative forms of engagement.

The spectrum describes five progressively increasing levels of participation: Inform, Consult, Involve, Collaborate, and Empower. These levels range from providing information to support understanding, through obtaining feedback and active participation in decision‐making processes, to shared partnership and ultimately public control over final decisions.

For this review, engagement activities were mapped to the five IAP2 participation levels using the framework's participation goals, public promises, and example engagement activities [50]. Studies were coded as *Inform* when parents participated only as recipients of an intervention or as research participants, with no reported role in the design, development, implementation, or evaluation of the research. *Consult* was assigned when parent views, experiences, or feedback were sought (e.g., through surveys, interviews, or focus groups) but final decisions remained researcher‐led. *Involve* was assigned when parents were engaged across multiple stages of intervention or research development, ensuring that their experiences and perspectives consistently informed the work (e.g., through workshops, or advisory activities). *Collaborate* was assigned when parents acted as partners in developing, refining, or selecting intervention components or research processes, such as through co‐design activities, advisory roles, or stakeholder working groups, and where there was evidence that parents contributed to identifying or shaping the preferred solution. *Empower* was reserved for studies in which final decision‐making authority was delegated to parents or parent groups.

For each included study, the IAP2 level was coded based on the role parents played across the research cycle, as described by the study authors, including intervention development, study design, implementation, evaluation, and decision‐making activities.

#### 2.4.3 Categorising Variables

To allow comparisons between heterogeneous studies, charted variables with free text descriptions (e.g., description of parental role or outcome measures) were assigned codes. Variable codes were iteratively developed using the constant comparison method [55]. The two reviewers generated the codes (TP and NH), compared them with the data until no new codes or insights emerged, agreed upon their definitions and applied them to the whole data set. For example, the ‘outcome measure’ for each article was charted as stated by the authors. To identify how these outcomes related to parents, each outcome was categorised using an iteratively developed code, for eaxmple, ‘parental knowledge’ or ‘prescription rates’. The definitions used are presented in Supporting Information: File 3.

To understand the specific role of parental engagement in relation to the described interventions, codes were assigned as described above (see Table 9 for definitions).

Results were tabulated to describe and summarise the findings according to the key conceptual categories listed in the charting table. Frequency tables were used to identify patterns in the data and determine gaps in the evidence.

## 3 Results

Search results are presented in Figure 3, with 111 articles meeting the criteria for analysis. There was substantial initial agreement between coders based on the inclusion criteria for full‐text articles within the database searches (*k* = 0.80).

**Figure 3:**
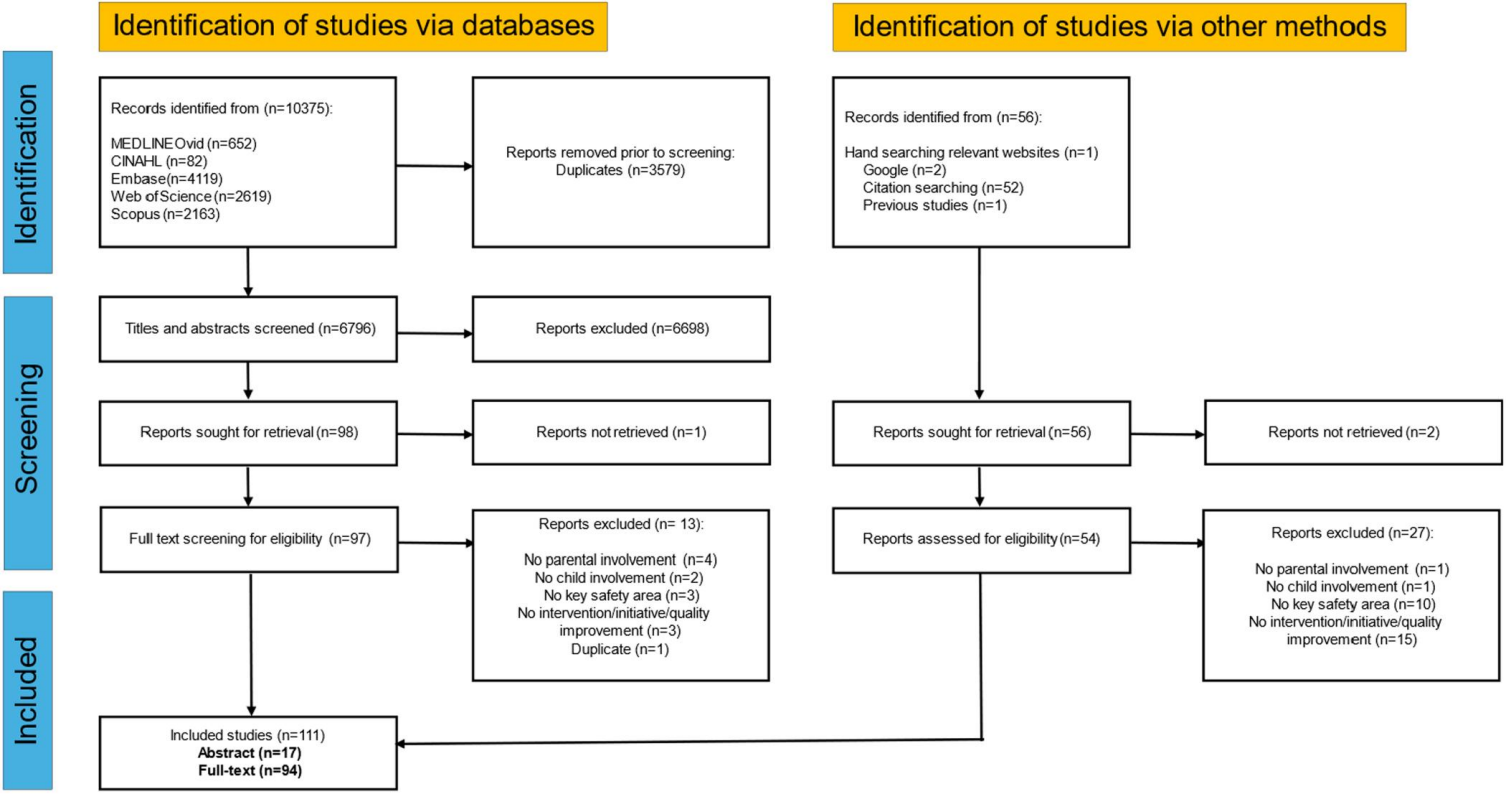
PRISMA diagram for included studies.

### 3.1 Characteristics of Included Studies

Characteristics of the included studies are detailed in Supporting Information: File 4. Most studies took place in North America and Western Europe, including full‐text articles (*n* = 94, 85%) and abstracts (*n* = 17, 14%). The care setting was most frequently hospital‐based care (*n* = 89, 80%) with only 19 studies (17%) conducted in primary or community care, or in both community and hospital‐based care (*n* = 2,2%).

Studies addressed one or more key safety area (Table 1). Communication and medication concerns were addressed most frequently (*n* = 72, 65% and *n* = 55, 50%, respectively), followed by documentation (*n* = 11, 10%), administrative incidents (*n* = 10, 9%) and diagnosis and assessment (*n* = 8, 7%).

**Table 1:** Key safety area addressed by interventions. Studies could be assigned multiple safety areas (*n* = 156).

Safety area	*N* = 156	% of included studies (*n* = 111)
Communication incidents	72	64.9
Medication incidents	55	49.5
Documentation incidents	11	9.9
Administrative incidents	10	9.0
Diagnosis incidents	8	7.2

A variety of study designs were undertaken, most commonly experimental designs, for example, randomised control trials (RCTs) (*n* = 43, 39%) and quality improvement projects (*n* = 36, 32%) (Table 2). Fewer studies were observational, for example, cohort studies (*n* = 23, 21%), or ‘other’, such as feasibility or pilot studies (*n* = 9, 8%).

**Table 2:** Study designs of included articles.

Study design	*N* = 111	%
Experimental, e.g., randomised control trial, quasi experimental, interventional, pre/post interventional, experimental	43	38.7
Quality improvement project	36	32.4
Observational, e.g., qualitative, prospective, descriptive, cohort, cross‐sectional, observational, time series	23	20.7
Other, e.g., validation study, design/development, pilot study, feasibility study	9	8.1

### 3.2 Interventions Identified

Interventions were categorised into 17 groups based on the key component of the intervention (Table 3). A third of studies incorporated more than one listed intervention category (*n* = 35, 32%), totalling 179 interventions across the studies. The most common intervention types included standardising processes (*n* = 26, 23%), such as handover checklists, and educational interventions aimed at the parents (*n* = 25, 23%), such as teaching parents about signs and symptoms to look for at home or training on dolls to administer medication. Written information was commonly used (*n* = 24, 22%), such as handouts and leaflets, and providing audio‐visual information (*n* = 21, 19%), such as pictograms or posters. More unique interventions included integrating a family member as an advisor on the improvement team undertaking the study (*n* = 1).

**Table 3:** Study intervention categories with examples. One or more interventions could be included per study (*n* = 179).

Categories	Interventions	*N* = 179	% of included studies (*n* = 111)	Examples
Education *N* = 85	Parent education	25	22.5	Consultation, training on dolls, teaching, ‘parent champion’, brief explanations
	Written information or instructions	24	21.6	Handouts, communication bundle, booklet, SMS texts
	Audio or visual information	21	18.9	Pictograms/diagrams/visual aids/posters/sliding wheel, videos
	Healthcare provider education	15	13.5	Teaching, leaflets
Processes *N* = 32	Standardisation of a process/framework	26	23.4	Handover, transition of care, written information, bedside verbal report, checklists, standard operating procedure
	New service	5	4.5	Peer to peer service, clinic for young parents of children
	Proactive risk assessment	1	0.9	FMEA (failure modes and affect analysis)
Communication *N* = 28	Communication techniques/counselling strategies	11	9.9	Teach‐back
	Families actively present (+/‐ participate)	7	6.3	During an assessment, a ward round, or discharge planning
	Communication tool	4	3.6	‘Patient safety menu’, Chloe's card*, column on whiteboard for parents
	Family‐activated care	3	2.7	Rapid response calls
	New ward round	3	2.7	Comfort round, nighttime communication intervention
Technology *N* = 19	Electronic interface	8	7.2	ParentLink**, mobile kiosk, equipment‐assisted interpreters, patient/parent EHR portal
	Web‐based	7	6.3	Support tool/digital animation/education/game/medication scheduler/pager
	Mobile application	4	3.6	Multiple apps developed
Parent perspectives *N* = 15	Gaining the parent perspective	14	12.6	Regarding transitions of care/discharge preferences, medical history by pharmacist, follow‐up phone calls or visits by nurses, nurse negotiation tool
	Family advisor on improvement team	1	0.9	Integrated member of the team

Abbreviations: EHR, Electronic Health Record; SMS, short message service.

* Chloe's card = a physical card to prompt parental communication.

** ParentLink = an interface for parents to share patient information, e.g., symptoms or medication history.

### 3.3 Outcome Measures

Thirty separate outcome measures were reported across all 111 papers, often with more than one outcome being measured (*n* = 161, Table 4). Definitions used when assigning outcome measures are provided in Supporting Information: File 3.

**Table 4:** Reported study outcome measures. One or more outcomes could be measured per study (*n* = 161).

Categories	Outcome measures	*N*	% (of 111)	% (of 161)
Parent‐specific *N* = 95	Parental perception	38	34.2	23.6
	Parental knowledge	21	18.9	13.0
	Parental dosing accuracy	11	9.9	6.8
	Seeking repeated medical review	6	5.4	3.7
	Parent medication administration	5	4.5	3.1
	Parental intervention use	9	8.1	5.6
	Parental awareness of intervention	1	0.9	0.6
	Parental stress	1	0.9	0.6
	Work or school days missed	1	0.9	0.6
	Timely access to medication	1	0.9	0.6
	Medication adherence	1	0.9	0.6
Healthcare provider‐specific *N* = 40	Healthcare provider perception	14	12.6	8.7
	Healthcare provider adherence to intervention	9	8.1	5.6
	Prescription rates	6	5.4	3.7
	Communication rate	3	2.7	1.9
	Healthcare provider knowledge	2	1.8	1.2
	Medication reconciliation errors	2	1.8	1.2
	Healthcare provider medication administration	1	0.9	0.6
	Staff awareness of intervention	1	0.9	0.6
	Medical history completeness	1	0.9	0.6
	Prescription error	1	0.9	0.6
Intervention outcomes *N* = 12	Intervention feasibility	7	6.3	4.3
	Validity or concordance against standard practice	3	2.7	1.9
	Intervention sensitivity/specificity	1	0.9	0.6
	Clinical safety	1	0.9	0.6
Other *N* = 14	Medical error or incident	5	4.5	3.1
	Primary outcome not stated/not measurable	5	4.5	3.1
	Length of stay	2	1.8	1.2
	Prototype development	1	0.9	0.6
	Mortality	1	0.9	0.6

Parent‐specific outcome measures were the most reported (*n* = 95/161, 59%), including parent perception (*n* = 38/111, 34%), parent knowledge (*n* = 21/111, 19%) and ability to accurately dose medication (*n* = 11/111, 10%). Healthcare provider‐specific outcomes (*n* = 30/161, 19%) focused on staff perception (e.g., of the intervention or a safety issue) (*n* = 14/111, 13%) and adherence to the intervention (*n* = 9/111, 8%). Few studies measured a change in relation to the intervention itself, for example, sensitivity or feasibility (*n* = 12/111, 11%). For example, the RCTs (*n* = 22) primarily used parent dosing accuracy, parent knowledge and perception, or prescription rates as outcome measures.

Where studies demonstrated a change in a parent‐specific outcome, e.g., knowledge, there was little evidence that this translated into an improvement in care quality or patient safety, although this was often stated. For example, Porter et al. [56] measured the validity of parent‐reported medications via an electronic kiosk in the emergency department, which improved subsequent physician documentation of their child's asthma medications. Although it can be inferred that this will improve safety efforts and care provided, outcomes relating to medication safety incidents or re‐admissions, for example, were not measured. Few studies reported outcome measures more specifically related to safety, such as mortality [57] or safety incidents related to care [58, 59, 60, 61, 62].

### 3.4 Reported Effectiveness

Overall, most studies reported a positive or unchanged effect of the intervention described (Table 5). Mixed outcome measure results were reported for some studies. For example, Wu et al. [58] developed a quality improvement collaborative for ward discharge processes with positive outcomes including a significant decrease in discharge‐related care failures, (34%–21%, *p* < 0.05) but with a worsening of the 30‐day re‐admission rate (4.5% vs. 6.3%, *p* = 0.05).

**Table 5:** Reported effectiveness of intervention outcomes measures. Outcome effectiveness was classified based on the author's description of the outcome in relation to their intended aims +/− any statistical analysis.

Outcome	Positive	Neutral	Negative
Parent knowledge	19	1	0
Healthcare knowledge	1	0	0
Errors (reduction of)*	20	3	0
Mortality or severe adverse events	1	1	0
Parent satisfaction	15	2	0
Healthcare satisfaction	4	0	0
Prescriptions (reduction of)	3	3	0
Information completeness	3	0	0
Intervention assessed	23	5	1
Intervention used by parent	2	1	0
Intervention used by healthcare	6	0	0
Communication	7	0	0
Parental involvement in care	2	0	0
Parental anxiety (reduction of)	1	0	0
Development of intervention	2	0	0

* The term ‘error’ has been used to reflect how the outcomes were described within the articles.

Lemiengre et al. [63] employed a brief intervention around parental concerns and safety netting with point of care c‐reactive protein (POC CRP) testing, aiming to reduce antibiotic prescription rates. They found the use of POC CRP did not affect prescription rates and the brief intervention increased antibiotic prescribing. The reasons behind this were considered to relate to the parental concerns influencing the physician's decision, choosing to then prescribe antibiotics.

Examples of interventions resulting in minimal impact or no change include Porter et al. [56] who showed no impact of their parent intervention (an electronic interface) on medication errors. Taylor et al. [64] reported their educational intervention for parents did not affect antibiotic prescribing rates, postulating that interventions only targeting parents may not have much effect.

### 3.5 Parent Engagement Within the Healthcare Intervention (PFE Framework)

Almost all included studies demonstrated parental engagement at the Direct Care level (*n* = 108/111, 97.3%), with only three studies (2.7%) engaging parents at an Organisational Design and Governance level, and no parents were engaged at a Policy Making level (Table 6). Across the continuum of engagement, the extent of parental engagement was most commonly classified as Consultation (*n* = 78, 70.3%), characterised by a predominantly healthcare provider‐led exchange of information, with parents able to ask questions and express concerns, but with limited influence over decision‐making. This was followed by Involvement (*n* = 32, 28.8%) and only one study (0.9%) demonstrating Partnership and Shared Leadership.

**Table 6:** Distribution of included studies across the carman patient and family engagement framework levels and continuum of engagement.

Level	Continuum		
	Consultation	Involvement	Partnership and shared leadership
Direct care	78	29	1
Organisational Design and Governance	0	3	0
Policy making	0	0	0

Whilst parental engagement at the Direct Care level was most commonly consultation‐based (*n* = 78), the few studies situated at the Organisational Design and Governance level (*n* = 3) were all classified as Involvement, characterised by more active parental engagement, including bidirectional information exchange and the incorporation of parental perspectives into decision‐making processes.

### 3.6 Parent Engagement in Research Activities (IAP2 Spectrum)

Most studies were classified at the lowest end of IAP2 Spectrum, with Inform being the most frequently identified category (*n* = 66, 59.5%) (Table 7), meaning parents within these studies were not engaged during intervention development or research activities. Few studies reached higher levels of research engagement, with 15 studies classified as Involve (13.5%) and nine studies as Collaborate (8.1%) [59, 65, 66, 67, 68, 69, 70, 71, 72].

**Table 7:** International association for public participation (IAP2) spectrum categories.

IAP2 category	*N*	%
Inform	66	59.5
Consult	21	18.9
Involve	15	13.5
Collaborate	9	8.1
Empower	0	0.0
Total	111	100

Each study's associated PFE and IAP2 Spectrum categories are provided in Supporting File 4.

### 3.7 Exemplars of High Parental Engagement in Research Activities

Studies with the highest ranking for parental engagement according to the IAP2 Spectrum (e.g., Collaboration, *n* = 9) were reviewed collectively to identify commonalities that could inform recommendations for future studies. A summary of how parents were engaged in these studies is provided in Table 8.

**Table 8:** Exemplar articles with high parental engagement in research activities.

Article	Intervention	PFE framework		How were parents engaged in the intervention?	IAP2 spectrum	How were parents engaged in the research activity?
		Level	Continuum			
Khan et al. [59]	Patient and Family Centered I‐PASS intervention (supporting structured communication during rounds)	Direct care	Involvement	Rounds were redesigned to be more family‐centred and improve communication. Parents were actively engaged in ward rounds by sharing concerns, contributing to care discussions, and confirming their understanding of the care plan through a structured read‐back process.	Collaborate	Families of paediatric patients were involved at all stages of the research process. This included designing the study, developing the family safety reporting method, refining data forms, co‐producing the intervention, interpreting findings, and writing the manuscript to disseminate the intervention and findings.
Goggin et al. [65]	Parent education and clinician communication intervention	Direct care	Involvement	Parents viewed a brief educational video about appropriate antibiotic use, rating their interest in receiving antibiotics for their child, and, in the higher‐intensity intervention, receiving a personalised educational brochure designed to support communication and shared decision‐making with clinicians during the consultation.	Collaborate	Worked closely with a multicultural group of parents. A Parent Research Associate was recruited as a core member of research team, contributing to all decisions and co‐leading the Community Advisory Board. Conducted parent focus groups and interviews during early planning and design stages.
Czulada et al. [66]	Inclusion of a family advisor on an improvement project team	Organisational design and governance	Involvement	A family advisor partnered with the improvement team to co‐develop a standardised communication policy that actively engaged parents in their child's care through regular updates, opportunities to discuss care plans and goals, and providing information on how to participate in their child's care.	Collaborate	The study was initiated after listening to parent feedback and concerns. The family advisor was trained as a researcher with the quality improvement team. They were part of the ICU clinical operations leadership group which set annual improvement goals and assigned associated project teams. They were also part of the multidisciplinary frontline improvement team and were trained in Lean Six Sigma.
Jolliff et al. [67]	Mobile health application (mHealth)	Direct care	Involvement	The intervention increased family caregivers' ability to coordinate, monitor, and manage their child's care through medication tracking, shared information, and communication tools across the caregiving network.	Collaborate	Followed a five‐stage, multi‐perspective co‐design process where end‐user representatives, including eight family caregivers, were active members of the design team.
Liang et al. [68]	Peer‐to‐peer family engagement consultation service	Direct care	Involvement	Parents were supported through peer‐to‐peer consultation which enhanced their ability to navigate services, communicate with healthcare professionals, advocate for their child, and participate in their child's care.	Collaborate	The Family Engagement Consultation Service was developed collaboratively with hospital staff and parent volunteers, with parents heavily involved in the design and implementation of the intervention. This included recruiting parent volunteers to pilot a peer‐to‐peer mentoring programme for hospitalised families and being provided additional training. Volunteers given a semi‐professional role as engagement consultants.
Khan et al. [69]	Family‐centered nighttime communication Intervention (communication bundle)	Direct care	Involvement	Parents participated in structured overnight communication with clinicians, receiving updates and contributing to discussions about their child's care to improve shared understanding.	Collaborate	Parents worked as active partners in the study from the start. Parents provided feedback during the intervention development, developed and tested the surveys used and helped iterate and test the intervention.
Marino et al. [70]	Chloe's card (parent‐centred communication tool)	Direct care	Involvement	Parents were supported to actively participate in communication and decision‐making discussions about their child's care through a parent‐centred communication tool.	Collaboration	Parents were engaged as partners throughout the design process, contributing to problem identification, idea generation, iterative refinement, and evaluation of the communication tool. The intervention was co‐designed and co‐created with parents, who helped develop alternatives and shape the final solution.
Walsh et al. [71]	Parent‐engaged proactive medication risk assessment	Organisational design and governance	Involvement	Parents participated in a medication‐safety risk assessment process, identifying home oral chemotherapy error risks and contributing to the development of strategies to improve the safety of medication management for children receiving cancer treatment.	Collaboration	Parents were members of the Failure Mode and Effects Analyses (FMEA) team, contributing to process mapping, identification and prioritisation of failure points, and the co‐development of risk‐reduction strategies that informed the study findings and proposed interventions.
Walsh et al. [72]	Web‐based home medication support tool	Direct care	Involvement	Parents used a family‐centred home medication management intervention to support communication, coordination, and safe administration of oral chemotherapy within the home setting.	Collaborate	Parents were included in the development team and as advisors, contributed to identifying intervention components, reviewed and iteratively refined prototypes, and participated in approval of the final intervention design.

Abbreviations: IAP2, International Association for Public Participation; PFE, Patient and Family Engagement.

### 3.8 Parental Roles

The specific roles parents played in relation to the interventions, and associated definitions used, are detailed in Table 9.

**Table 9:** The parent role in relation to the interventions, inclusive of being part of the intervention and/or the design or implementation of the intervention, with associated definitions. One or more parental roles could be assigned (*n* = 176).

Parent role	Definition	*N* = 176	% of included studies (*n* = 111)	Examples of interventions
Education	Received verbal education or educational resources (beyond simple instructions, e.g., on dosing medications)	42	37.8	Educating parents using pictograms or about antibiotic prescribing
Part of clinical activity	The parents contributed to or were present for a clinical activity which they were not previously part of, such as ward rounds, giving a history, medication history or the discharge process	36	32.4	Family Centred Rounds, parent present for clerking with mental health nurse, medication reconciliation
Use new tool	A new tool, including new devices, mobile applications or pieces of equipment, is given to the parents and the parents use this to complete a task	30	27.0	Dosing syringes, self‐triage app, inpatient health portal, educational resources (e.g., handouts and online)
Design	Parent was involved in the design of the study or intervention, which includes the initiation of the project	29	26.1	Family Centred Rounds, online medication tool, handover frameworks, educational resources
Parent performance assessed (for a given activity)	The parent had some aspect of a practical activity assessed by an HCP	21	18.9	Liquid medication dosing, asthma medication plan comprehension, antibiotic usage education, discharge information
Qualitative data collection	Parents are interviewed where their opinions and/or information is gathered by the research team	8	7.2	Chloe's card, family centred rounds, interactive respiratory disease booklet
Accessing a new service	A new service is created for parents who are able to access this on behalf of their children for healthcare input such as outreach clinic or telemedicine services. A new service should be completely distinct from old services rather than an additional layer of care in the existing procedures	5	4.5	Peer to Peer service, clinic for young parents of children
Delivery	Parents involved in the formal roll‐out of the project, for example, teaching staff or delivering a service	2	1.8	Peer‐to‐Peer support service, I‐PASS intervention for rounding
Generating ideas	Related to activities of using parents to generate suggestions or new interventions for testing/mitigation of harm	2	1.8	FMEA for oral chemotherapy administration, checklist development
Given new role	Parent is given a new role or responsibility in the care/management of their child or within the clinical team/activity	1	0.9	Parent activation of Rapid Response Calls

Parents were most frequently the target of an educational intervention or provided with educational resources (*n* = 42, 38%). Parents were regularly part of a clinical activity (*n* = 36, 32%), such as family‐centred rounds or medication reconciliation, or were asked to use specific equipment or a new tool that had been developed (*n* = 30, 27%), e.g., a self‐triage mobile application. Parents were engaged in supporting the design of the study or the intervention in over a quarter of studies (*n* = 29, 26%) and their opinions sometimes sought on the intervention (*n* = 8, 9%) but were seldom asked to generate new ideas for change (*n* = 2, 2%). Nearly a fifth of studies assessed the parental performance of a task (*n* = 21, 19%), including dosing liquid medications or understanding a management plan. Less frequently the intervention gave parents a new role within the system (*n* = 1, 1%) or parents were an active part of delivering the intervention to others (*n* = 2, 2%), such as a peer‐to‐peer support service.

### 3.9 Health Inequalities in Research Participation

There was a mixed picture when studies considered parents and healthcare users as participants from a range of backgrounds. For example, non‐English‐speaking participants were excluded from many of the studies, whereas Kosack et al. demonstrated the benefits of ensuring an interpreter is present to improve communication and family participation [73].

Some studies identified that parents with low health literacy or educational status had a greater risk of contributing to a safety incident, for example a ‘medication error’ [71, 74]. Studies aiming to mitigate the risks associated with low health literacy, for example Yin et al., reported that the benefits of such interventions (e.g., pictogram instructions) have varying levels of success when divided into subgroups based on health literacy, with the intervention having no effect on participants with ‘adequate’ health literacy [75].

Yin et al. also demonstrated that parents who were provided with low‐literacy asthma management plans were less likely to make an error in relation to their child's medication compared to those given the standard plan [76]. Whereas one study excluded participants who did not understand the given task within the intervention, relating to administering epilepsy medication at home, despite acknowledging that these medications pose a high‐risk of handling errors [77]. This suggests interventions require a more targeted approach, with special consideration given to include those at higher risk of being involved in a safety incident.

Other studies took health literacy into consideration, for example, by adjusting for health literacy in their analyses [78], by ensuring staff were aware of health literacy as part of an educational intervention [79], and involving health literacy experts on the intervention team [58]. Further examples included standardising resources for parents with low and adequate literacy levels [80], educational e‐platforms available in more than one language [81, 82], capturing language preferences at the emergency department front door to integrate into the electronic medical record [83], increasing interpreter availability [84, 85, 86] and heath literacy toolkits to improve communication [87].

Schopf et al. [88] evaluated a system change for patients with autism spectrum disorder or intellectual disabilities, where a nurse would attend the emergency department to triage and assess the patient, rather than wait for them to arrive on the ward. This better utilised family members who were otherwise too tired or had gone home when the patient arrived on the ward. This study provides a good example of quality improvement efforts attempting to amend the work system (and its pathways) to accommodate the person factors (e.g., needs and characteristics) of this patient group and their families.

## 4 Discussion

A wide variety of interventions aiming to improve paediatric safety with some degree of parental engagement has been described. Interventions ranged from standardising clinical processes involving parents, ensuring parents are present during clinical management decisions, employing electronic interfaces and communication tools, to incorporating parents as family advisors on the research team. However, the most common types of intervention identified were educational in nature, primarily aimed at educating the parents in clinical activities or tasks, such as how to dose and administer medication.

Outcome measures were mostly parent‐specific, assessing how the interventions changed the parents' perception, knowledge or ability to correctly perform a given task. Outcomes measures related to the intervention itself, such as feasibility, were less commonly assessed, and outcomes related to impact on quality and safety in the real world were rare.

Parents played a wide variety of roles, but the predominant type of engagement in relation to both study design or intervention development and as research participants was at the lower end of both the PFE framework (e.g., direct care and consultation) and the IAP2 Spectrum (e.g., inform). Parents being engaged as partners in care planning, service design, governance activities, or research collaboration was much less frequent, with the parental role remaining largely tokenistic in nature, where the part they play provides “no assurance of changing the status quo” [89]. This is in keeping with well‐documented concerns relating to wider patient engagement efforts and how tokenism results in the lack of genuine influence over decision‐making [90].

Although multiple positive benefits of parental engagement were described by authors, these were anecdotal in nature. Reflections or evaluations on engaging parents, including the perceived advantages and challenges, were rare and not formalised.

### 4.1 Parental Engagement in the Research Process

Examples were identified of parents being successfully engaged across different stages of the research cycle and within quality improvement initiatives (Table 8). However, most included studies engaged parents at the lower levels of the IAP2 Spectrum, with over half classified as Inform, indicating that parents were engaged only as recipients of interventions or as research participants, with no reported role in the design, development, implementation, or evaluation of the research.

Flynn et al. reported that child and family engagement was most commonly characterised at the Consult and Involve levels, with only a small number of studies demonstrating engagement in leadership or support roles within research teams [54]. Crowther et al. similarly found that children, young people, and parents were inconsistently engaged across the research cycle, with engagement most commonly occurring during the design of research methods and intervention development rather than throughout all stages of research [91].

Parents could play a much greater role across the co‐production cycle, including co‐assessing, co‐deciding, co‐designing and co‐delivering interventions [10]. This would create multiple opportunities for meaningful engagement and participation, ensuring the goals of the intervention align with the priorities of those who will experience care [10].

The facilitators, barriers and challenges associated with engaging parents as co‐researchers have previously been described in detail [6, 91, 92]. Several of these challenges were reflected within the included studies, where additional staff and resources were required to maintain higher levels of engagement [65]. Parental engagement should therefore not be considered a single aspect for individual projects but viewed as a dynamic process that requires nurturing to remain active and meaningful [92]. Achieving meaningful and sustained engagement is likely to require an early engagement plan to ensure that the necessary resources, infrastructure, and funding are available throughout the research process.

### 4.2 Effects of Engagement on Study Design and Delivery

Multiple studies briefly commented on the perceived benefits and drawbacks of engaging parents within their research and interventions. This was most evident within the higher‐level engagement studies where the added value of parental engagement was reported as improving the quality of the data, being integral to having a full understanding of the family perspective and adding indispensable value to the design and implementation of the study [59, 65, 67]. But these impacts were not formally evaluated, which was acknowledged [68], in keeping with previous reviews evaluating parental engagement in healthcare interventions [54, 91].

Previously documented benefits of parental engagement include the development of more sustainable and population‐appropriate interventions, increasing the quality of results based on holistic experiences, and empowering parents through increasing their confidence and research skills [6]. This evidence is captured primarily through informal qualitative feedback or observations by the study team [6, 91].

A deeper understanding of how parental engagement influences the wider research process, and not just its primary or secondary outcomes, would enable future researchers to make more informed decisions about how best to incorporate parents in study design and intervention development. Formalising parental engagement within research processes, including establishing standardised expectations for evaluating and reporting its effects, would support this effort. Achieving this is likely to require sustained commitment from both organisational leaders and researchers to drive such change [93].

### 4.3 Effects of Engagement on Care Quality and Safety

The review findings indicate that parental engagement in paediatric safety interventions was overwhelmingly concentrated at the Direct Care level, with only three studies demonstrating engagement in Organisational Design and Governance and none at the Policy‐Making level. This suggests that parents were primarily utilised to support the delivery of care for their own child and may be underutilised in shaping the systems, services, governance structures, and policies intended to improve paediatric safety.

Across the continuum of engagement, parental engagement was most commonly characterised by Consultation rather than Involvement or Partnership and Shared Leadership. Parents were therefore most often engaged through activities such as receiving information, asking questions, expressing concerns, and providing feedback, with comparatively limited influence over care planning, service improvement, or decision‐making processes.

Similar patterns have been reported elsewhere. In a review of patient safety interventions in primary care, Pang et al. found that family member engagement was uncommon and, where present, was largely limited to lower levels of engagement [8]. Likewise, Seyfulayeva et al. found that patient and family engagement in perioperative safety initiatives was predominantly situated at the Direct Care level and most commonly adopted a consultation‐based approach, with interventions primarily focused on patient information, education, and communication [52].

Patient and family engagement, positioning them as active partners in care decisions to create safer healthcare systems, has been identified as a priority by the WHO Global Patient Safety Action Plan [9], and as a key element for setting up agendas across multiple levels of paediatric patient safety [94]. Despite the need to understand how engagement affects research [95], evaluating and measuring its impact remains challenging. This is partly due to difficulties in establishing causal links between engagement and outcomes of interest, and the reliance of many evaluation tools on perceived rather than objective benefits [96]. It is therefore unclear from the data whether an increase in parent knowledge or ability, for example, translates into safer paediatric care.

For researchers and organisations to meaningfully engage parents as part of the solution to an identified issue (i.e., as a research participant or within the research development) and promote the parent voice, we need consensus on how to best demonstrate the evidence of parental contribution and influence when they're adequately engaged. This does not necessarily mean the demonstration of impact on outcome measures but across the whole process.

Dukhanin et al. argue the evaluation of engagement is inconsistent and underdeveloped, consequently proposing a comprehensive taxonomy of outcome and process measures for engagement in healthcare decision making [96]. The taxonomy distinguishes between different types of process metrics, for example. These include direct measures (e.g., setting the agenda), surrogate measures (e.g., attendance of engagement participants), and more abstract aggregate measures (e.g., respect and trust). Such a framework offers a useful structure to be adopted or adapted by researchers when designing, implementing, and evaluating engagement activities.

Proxy measures for safety, such as parental knowledge, should be carefully considered and used in conjunction with parent‐reported safety measures to more accurately capture parental influence on safety.

Evaluations should also include an exploration of why some interventions with parents as research participants result in negative outcomes. For example, the study from Lemiengre et al., [63] where their brief intervention aiming to reduce antibiotic prescribing found parent concerns likely influenced the physician's decision and subsequently increased antibiotic prescribing, provides an example of where multiple factors need to be considered and explored when implementing and evaluating interventions (e.g., via a systems approach, see below). For example, the person factors (e.g., their characteristics and needs) associated with the complex interactions between parent concerns and physician decision‐making (or shared decision‐making) need to be considered in addition to how providing further information or objective testing can impact outcome measures.

### 4.4 A Systems Approach Is Needed

Most studies targeted their interventions towards parents, with a focus on changing one or more parent characteristics, e.g., knowledge, to enable change. Complexity arises from the decision‐making process in paediatric cases being inherently contextual and relational (between the child, parents and healthcare staff) [28]. Targeting single system elements, such as people or equipment [97], does not account for the interacting relationships between the people and other elements of the system, from which safety performance and other outcomes in complex systems derive [98]. Therefore, parents should not be the only focus of change but considered part of a multi‐faceted intervention targeting multiple factors amenable to improvement. A systems‐approach would also support the re‐framing of how parents were viewed within some articles, where the responsibility for making mistakes and contributing to subsequent harm was sometimes considered to lie with the parent [74, 77].

Engaging parents to a greater extent in the study design and conduct may help to tackle these concerns as well as democratising research, thereby supporting its relevance and quality through making it more accountable, legitimate and inclusive [99]. Meaningful engagement may therefore help to redress the aforementioned inequalities associated with parents as research participants.

The actions of parents can be vital safeguards within our healthcare systems [25, 29]. How their engagement affects research activities and healthcare processes requires further exploration. The aim here should not be to just measure the impact parents have on research outcomes, but an exploration of the role parents play within the systems under scrutiny, and the outcomes that emerge as a result. Meaningful engagement activities could be utilised to consider important contextual parent characteristics and needs, the influence these have on their interactions with the surrounding system elements, and how these interactions can be optimised to improve safety, performance and wellbeing [98]. Such a human‐centred approach would support the co‐design of an intervention or system change with parents, through taking their characteristics (e.g., capabilities, limitations) into account early on and throughout the process.

### 4.5 A Dearth of Primary Care Research

Few studies (*n* = 19, 17%) were based within primary or community care settings, consistent with the broader patient safety research focus on hospital‐based specialities [100]. Pang et al. [8] only identified 12 RCTs for patient safety interventions within primary care with PFE. Consistent with the results of this study, these RCTs mostly focused on medication safety, had little effect on outcome measures, and demonstrated minimal levels of patient or family engagement.

There is a need for primary care researchers and organisations to do more to co‐develop, implement and measure safety interventions with parents to improve paedatric care delivery, or consider how to adapt the hospital care‐based interventions listed in this review.

### 4.6 Strengths and Limitations

The search strategy was comprehensive, following established methodological guidance, and informed by empirical data to prioritise patient safety areas of interest where parents can influence care [29]. Some key safety areas, therefore, were not included, and data are missing from these areas, for example, incidents relating to investigations. There was a predominant focus on medication and communication safety within the studies, but this is likely reflective of the wider focus on these areas within the field of patient safety [31, 101].

Previous reviews employing the PFE framework have reported that further definitions are needed for each group, to allow more accurate classification of interventions [52]. Although this concern was discussed amongst the reviewers, it was felt that the benefit of the framework presenting more of a spectrum of engagement allowed for greater flexibility when categorising the studies. Given the heterogeneity of the data, this was a beneficial starting point of the analysis, generating rich discussions around how parents were included in each study.

Additionally, although multiple international studies were included, the review was restricted to publications in the English language, which may have introduced language bias and led to the exclusion of relevant studies published in other languages. Consistent with scoping review methodology [41, 43], no formal critical appraisal of included studies was undertaken. Therefore, the quality and risk of bias of the evidence base were not assessed, and findings should be interpreted as descriptive rather than evaluative.

## 5 Conclusion

Parental engagement is widely recognised as essential to improving paediatric patient safety. However, this review highlights that meaningful engagement remains limited, inconsistent, and often confined to passive or tokenistic roles across care settings. There is a clear need to move towards sustained, equitable partnerships with parents, both as active contributors to research and as partners in the delivery, design and improvement of healthcare services and safety interventions. Achieving this shift will require embedding parental engagement as a core component of research and quality improvement, alongside developing consensus‐driven, standardised approaches to evaluating and reporting engagement processes and their influence on outcomes. These efforts will be critical to enabling a more democratic, inclusive, and human‐centred approach to safety, in which parental perspectives are not only heard but meaningfully shape safer paediatric care, healthcare services, and policy.

## Author Contributions

**Thomas Purchase:** conceptualisation, investigation, funding acquisition, methodology, writing – original draft, writing – review and editing, formal analysis, project administration, data curation. **Nicholas Hodson:** writing – review and editing, formal analysis, investigation, data curation, visualisation. **Samuel Li:** investigation, writing – review and editing. **Isobel Joy McFadzean:** writing – review and editing. **Joanne Hughes:** formal analysis, writing – review and editing. **Philippa Rees:** conceptualization, writing – review and editing. **Fiona Wood:** supervision, writing – review and editing. **Sarah Yardley:** supervision, writing – review and editing. **Adrian Edwards:** supervision, writing – review and editing. **Andrew Carson‐Stevens:** conceptualization, supervision, methodology, writing – review and editing, funding acquisition.

## Conflicts of Interest

The authors declare no conflicts of interest.

## Permissions

Health Affairs has granted permission for Carman et al.'s Figure 1 to be reproduced in this article. The IAP2 International Federation has granted permission to reproduce the IAP2 Spectrum within the article.

## Supporting information


Supporting File

## Data Availability

The data are available upon reasonable request from the corresponding author.
