# Supplementary material for: Engaging Parents in Healthcare Interventions to Improve Paediatric Patient Safety: A Scoping Review
Source: Health Expect. 2026 Oct 12;29(5):e70917. doi: 10.1111/hex.70917 (PMC13655772; doi:10.1111/hex.70917)
Supplement: Supplementary file 1 [file HEX-29-e70917-s001.docx]

**Engaging parents in healthcare interventions to improve paediatric patient safety: a scoping review**

**Supplementary files**

Contents

**Pages 2-7**. Supplementary File 1. Database search strategies – Ovid Medline, EMBASE, CINAHL, Web of Science and Scopus

**Page 8**. Supplementary File 2. List of sites included in the hand search strategy

**Pages 9-10**. Supplementary File 3. Table S1. Outcome measures and associated definitions

**Pages 11-23**. Supplementary File 4. Table S2. Characteristics and key variables of included articles

**Pages 24-33**. Supplementary File 5. References for included articles

Supplementary File 1.

**Database search strategies – Ovid Medline, EMBASE, CINAHL, Web of Science and Scopus**

**Ovid MEDLINE** <1946 to Aug 31, 2025>

1	((vaccin* or immuni* or medicat* or triag*) adj3 error).mp.

2	((vaccin* or immuni* or medicat* or triag*) adj3 safety).mp.

3	((vaccin* or immuni* or medicat* or triag*) adj3 incident*).mp.

4	exp Adverse Drug Events/

5	exp Medication errors/

6	((clinical assessment or patient safety) adj3 error*).mp.

7	((clinical assessment or patient assessment) adj3 safety*).mp.

8	((clinical assessment or patient assessment) adj3 incident*).mp.

9	exp Diagnostic Errors/

10	((healthcare adj3 error*) or healthcare errors).mp.

11	exp Medical Errors/ or medical incident.mp.

12	((serious* or incident*) adj3 error*).mp.

13	((error* or failure* or incident* or patient safety) adj3 communic*).mp.

14	((error* or failure* or incident* or patient safety) adj3 advic*).mp.

15	((error* or incident* or safety*) adj3 admin*).mp.

16	((error* or incident* or safety*) adj3 patient record*).mp.

17	((error* or incident* or safety*) adj3 medical record*).mp.

18	((adverse* or safety*) adj3 event*).mp.

19	exp Patient Safety/

20	Child*.mp. or exp Child/

21	exp Adolescent/ or adolescen*.mp.

22	exp Infant/ or exp Infant, Newborn/

23	(teen* or youngster* or youth* or juvenile* or paediatri* or pediatri*).mp.

24	(Young adj2 (person* or adult* or individual*)).mp.

25	exp Quality Improvement/

26	intervention.mp.

27	(Improve* adj3 interven*).mp.

28	(safe* adj3 improve*).mp.

29	(Safe* adj3 intervent*).mp.

30	exp Safety management/

31	exp Risk management/

32	((error* or risk* or mistake* or adverse event) adj3 prevent*).mp.

33	((error* or risk* or mistake* or adverse event) adj3 reduc*).mp.

34	exp Parents/

35	guardian.mp. or Legal Guardians/

36	exp Family/

37	carer.mp. or exp Caregivers/

38	family involvement.mp.

39	parent involvement.mp.

40	1 or 2 or 3 or 4 or 5 or 6 or 7 or 8 or 9 or 10 or 11 or 12 or 13 or 14 or 15 or 16 or 17 or 18 or 19

41	20 or 21 or 22 or 23 or 24

42	25 or 26 or 27 or 28 or 29 or 30 or 31 or 32 or 33

43	34 or 35 or 36 or 37 or 38 or 39

44	40 and 41 and 42 and 43

45	limit 44 to (english language and yr="2000 -Current")

**Embase (via Ovid)**

All Embase subject headings were verified against Emtree when replicating the Ovid MEDLINE search strategy. The only amendment required was replacement of *adverse drug event* with the Emtree-preferred term *adverse drug reaction*.

1	((vaccin* or immuni* or medicat* or triag*) adj3 error).mp.

2	((vaccin* or immuni* or medicat* or triag*) adj3 safety).mp.

3	((vaccin* or immuni* or medicat* or triag*) adj3 incident*).mp.

4	exp Adverse Drug reaction/

5	exp Medication errors/

6	((clinical assessment or patient safety) adj3 error*).mp.

7	((clinical assessment or patient assessment) adj3 safety*).mp.

8	((clinical assessment or patient assessment) adj3 incident*).mp.

9	exp Diagnostic Errors/

10	((healthcare adj3 error*) or healthcare errors).mp.

11	exp Medical Errors/ or medical incident.mp.

12	((serious* or incident*) adj3 error*).mp.

13	((error* or failure* or incident* or patient safety) adj3 communic*).mp.

14	((error* or failure* or incident* or patient safety) adj3 advic*).mp.

15	((error* or incident* or safety*) adj3 admin*).mp.

16	((adverse* or safety*) adj3 event*).mp.

17	((error* or incident* or safety*) adj3 patient record*).mp.

18	((error* or incident* or safety*) adj3 medical record*).mp.

19	exp Patient Safety/

20	Child*.mp. or exp Child/

21	exp Adolescent/ or adolescen*.mp.

22	exp Infant/ or exp Infant, Newborn/

23	(teen* or youngster* or youth* or juvenile* or paediatri* or pediatri*).mp.

24	(Young adj2 (person* or adult* or individual*)).mp.

25	exp Quality Improvement/

26	intervention.mp.

27	(Improve* adj3 interven*).mp.

28	(safe* adj3 improve*).mp.

29	(Safe* adj3 intervent*).mp.

30	exp Safety management/

31	exp Risk management/

32	((error* or risk* or mistake* or adverse event) adj3 prevent*).mp.

33	((error* or risk* or mistake* or adverse event) adj3 reduc*).mp.

34	exp Parents/

35	guardian.mp. or Legal Guardians/

36	exp Family/

37	carer.mp. or exp Caregivers/

38	family involvement.mp.

39	parent involvement.mp.

40	1 or 2 or 3 or 4 or 5 or 6 or 7 or 8 or 9 or 10 or 11 or 12 or 13 or 14 or 15 or 16 or 17 or 18 or 19

41	20 or 21 or 22 or 23 or 24

42	25 or 26 or 27 or 28 or 29 or 30 or 31 or 32 or 33

43	34 or 35 or 36 or 37 or 38 or 39

44	40 and 41 and 42 and 43

45	limit 44 to (english language and yr="2000 - 2025")

**CINAHL**

((AB (((vaccin* OR immuni* OR medicat* OR triag*) N3 error))) OR (AB ((vaccin* OR immuni* OR medicat* OR triag*) N3 safety)) OR (AB ((vaccin* OR immuni* OR medicat* OR triag*) N3 incident*)) OR (AB Adverse drug event) OR ((MH "Adverse Drug Event+")) OR ((MH "Medication Errors+")) OR (AB ((clinical assessment OR patient safety) N3 error*)) OR (AB ((clinical assessment OR patient assessment) N3 safety*)) OR (AB ((clinical assessment OR patient assessment) N3 incident*)) OR ((MH "Diagnostic Errors+")) OR ((MH "Health Care Errors+")) OR (AB "medical incident*") OR (AB ((error* OR failure* OR incident* OR patient safety) N3 communic*)) OR (AB ((error* OR failure* OR incident* OR patient safety) N3 advic*)) OR (AB ((error* OR incident* OR safety*) N3 admin*)) OR (AB ((adverse* OR safety*) N3 event*)) OR ((MH "Patient Safety+"))) ((AB (( error* OR incident* OR safety* ) N3 "patient record*" )) OR (AB (( error* OR incident* OR safety* ) N3 "medical record*" )) OR ( ABS (("medical record*"))) AND (((MH "Child+") OR "child") OR ((MH "Adolescence+")) OR ((MH "Infant+") OR (MH "Infant, Newborn+")) OR (AB (teen* OR youngster* OR youth* OR juvenile* OR paediatri* OR pediatri*)) OR ((MH "Young Adult")) OR (AB (Young N2 (person* OR adult* OR individual*)))) AND (((MH "Quality Improvement+")) OR (AB intervention) OR (AB Improve* N3 interven*) OR (AB safe* N3 improve*) OR (AB Safe* N3 intervent*) OR ((MH "Risk Management+")) OR (AB ((error* OR risk* OR mistake* OR adverse event) N3 prevent*)) OR (AB ((error* OR risk* OR mistake* OR adverse event) N3 reduc*))) AND (((MH "Parents+")) OR ((MH "Guardianship, Legal+") OR "guardian*") OR ((MH "Family+")) OR ((MH "Caregivers")) OR (AB carer) OR (AB family involvement) OR (AB parent involvement))

**Web of Science**

#1 AB=((vaccin* OR immuni* OR medicat* OR triag*) NEAR error*)

#2 AB=((vaccin* OR immuni* OR medicat* OR triag*) NEAR safety*)

#3 AB=((vaccin* OR immuni* OR medicat* OR triag*) NEAR incident*)

#4 AB=("adverse drug event*")

#5 AB=(medication NEAR error*)

#6 AB=((clinical assessment OR patient safety) NEAR error*)

#7 AB=((clinical assessment OR patient assessment) NEAR safety*)

#8 AB=((clinical assessment OR patient assessment) NEAR incident*)

#9 AB=(diagnostic NEAR error*)

#10 AB=(healthcare NEAR error*)

#11 AB=("medical error*" OR "medical incident*")

#12 AB=((serious* OR incident*) NEAR error*)

#13 AB=((error* OR failure* OR incident* OR "patient safety") NEAR communic*)

#14 AB=((error* OR failure* OR incident* OR "patient safety") NEAR advic*)

#15 AB=((error* OR incident* OR safety*) NEAR admin*)

#16 AB=((error* OR incident* OR safety*) NEAR "patient record*")

#17 AB=((error* OR incident* OR safety*) NEAR "medical record*")

#18 AB=((adverse* OR safety*) NEAR event*)

#19 AB=("patient safety")

#20 AB=(child* OR adolescen* OR infant* OR newborn* OR teen* OR youngster* OR youth* OR juvenile* OR paediatri* OR pediatri* OR (young NEAR (person* OR adult* OR individual*)))

#21 AB=("quality improvement" OR (quality NEAR improvement*)) OR intervention* OR (improv* NEAR interven*) OR (safe* NEAR improve*) OR (safe* NEAR interven*) OR ("safety management" OR (safety NEAR management)) OR ("risk management" OR (risk NEAR management)) OR ((error* OR risk* OR mistake* OR "adverse event") NEAR prevent*) OR ((error* OR risk* OR mistake* OR "adverse event") NEAR reduc*))

#22 TS=(parent* OR guardian* OR "legal guardian*" OR famil* OR carer* OR caregiver* OR "family involvement" OR "parent involvement")

#23 #1 OR #2 OR #3 OR #4 OR #5 OR #6 OR #7 OR #8 OR #9 OR #10 OR #11 OR #12 OR #13 OR #14 OR #15 OR #16 OR #17 OR #18 OR #19

#24 #23 AND #20 AND #21 AND #22

**Scopus**

( ( ABS ( ( vaccin* OR immun* OR medicat* OR triag* ) W/3 error* ) ) OR ( ABS ( ( vaccin* OR immuni* OR medicat* OR triag* ) W/3 safety ) ) OR ( ABS ( ( vaccin* OR immuni* OR medicat* OR triag* ) W/3 incident* ) ) OR ( ABS ( "Adverse drug event*" ) ) OR ( ABS ( ( "clinical assessment" OR "patient safety" ) W/3 error* ) ) OR ( ABS ( ( "clinical assessment" OR "patient assessment" ) W/3 safety* ) ) OR ( ABS ( ( "clinical assessment" OR "patient assessment" ) W/3 incident* ) ) OR ( ABS ( healthcare W/3 error* ) ) OR ( ABS ( "medical error*" OR "medical incident*" ) ) OR ( ABS ( ( serious* OR incident* ) W/3 error* ) ) OR ( ABS ( ( error* OR failure* OR incident* OR "patient safety" ) W/3 communic* ) ) OR ( ABS ( ( error* OR failure* OR incident* OR "patient safety" ) W/3 advic* ) ) OR ( ABS ( ( error* OR incident* OR safety* ) W/3 admin* ) ) OR ( ABS ( medication W/3 error* ) ) OR ( ABS ( diagnostic W/3 error* ) ) OR ( ABS ( "patient safety" ) ) OR ( ABS ( ( adverse* OR safety* ) W/3 event* ) ) ) OR ( ( ABS ( ( error* OR incident* OR safety* ) W/3 "patient record*" ) ) OR ( ABS ( ( error* OR incident* OR safety* ) W/3 "medical record*" ) ) AND ( ( ABS ( child* ) ) OR ( ABS ( adolescen* ) ) OR ( ABS ( infant* ) ) OR ( ABS ( newborn* ) ) OR ( ABS ( teen* ) ) OR ( ABS ( youngster* ) ) OR ( ABS ( youth* ) ) OR ( ABS ( juvenile* ) ) OR ( ABS ( paediatri* ) ) OR ( ABS ( pediatri* ) ) OR ( ABS ( young* W/3 ( person* OR adult* OR individual* ) ) ) ) AND ( ( ABS ( "Quality improvement" OR ( quality W/3 improvement* ) ) ) OR ( ABS ( intervention* ) ) OR ( ABS ( improve* W/3 interven* ) ) OR ( ABS ( safe* W/3 improve* ) ) OR ( ABS ( safe* W/3 interven* ) ) OR ( ABS ( "safety management" OR ( safety W/3 management ) ) ) OR ( ABS ( "risk management" OR ( risk* W/3 management ) ) ) OR ( ABS ( ( error* OR risk* OR mistake* OR "adverse event" ) W/3 prevent* ) ) OR ( ABS ( ( error* OR risk* OR mistake* OR "adverse event" ) W/3 reduc* ) ) ) AND ( ( ABS ( parent* ) ) OR ( ABS ( guardian* OR "legal guardian" ) ) OR ( ABS ( famil* ) ) OR ( ABS ( carer* OR caregiver* ) ) OR ( ABS ( "family involvement" ) ) OR ( ABS ( "parent* involvement" ) ) ) AND PUBYEAR > 1999 AND PUBYEAR < 2025 AND ( LIMIT-TO ( LANGUAGE , "English" ) )

Supplementary File 2.

**List of sites included in the hand search strategy**

The search included

- JBI Evidence Synthesis
- Google scholar
- Prospero
- The Campbell Collaboration
- BMJ Quality & Safety
- Journal of Patient Safety
- The Joint Commission Journal on Quality and Patient Safety
- Journal of Patient Safety and risk management
- BMJ Open Quality
- Journal for Healthcare Quality
- Implementation Science

Supplementary File 3.

**Table S1. Outcome measures and associated definitions**. Where an outcome is not specifically stated we have taken the outcomes from their reported effectiveness.

| Outcome measure | Definition |
| --- | --- |
| Parental knowledge | Usually secondary to educational intervention where either knowledge can be measured or is used to assess or infer a secondary outcome. This can include knowledge / awareness / understanding of their rights and responsibilities, or how to complete a task. |
| Parental perception | Usually related to an intervention where the parents / caregivers are asked for opinions / feedback on a subjective state, i.e. safety or quality of an intervention, or including parent satisfaction with the intervention, or advice given as part the intervention (in the design or implementation phase) |
| Parental dosing accuracy | Usually involving methods or tools to improve dosing accuracy in parent’s dosing medication |
| Medication adherence | Usually related to the timings of, or non-omission of either ‘as needed’ or regular medications and techniques used to mitigate mistakes or incidents related to this |
| Non-parental | Outcome measure did not look at any aspect of parent / caregiver input / output. Not specifically measuring a parent outcome. |
| Healthcare provider perception | Usually related to an intervention where the staff are asked for opinions / feedback on a subjective state i.e. safety or quality of intervention or staff satisfaction |
| Prescription rates | Relating to the usage rates of prescription medications, e.g., antibiotics with interventions targeting either parents or HCPs, and inappropriate antibiotic treatment |
| Parental awareness of intervention | Relating to the assessment of parents being aware of the availability of the intervention(s) |
| Staff awareness of intervention | Relating to the assessment of staff being aware of the availability of the intervention(s) |
| Primary outcome not stated/not measurable | The primary outcome was not explicitly stated in the study or the design of the study such that no outcome could be measured |
| Intervention feasibility | Relating to an outcome where parents or staff opinion was taken specifically on feasibility of the usage or development of an intervention |
| Healthcare provider adherence to intervention | Relates to an intervention where HCPs are asked to perform a task and the completeness or number of tasks completed is reviewed  OR  Relating to the usage of the intervention as intended by the designers of the intervention |
| Parent medication administration | Relates to the process of giving medication, e.g., the route / rate (as opposed to the dosing and drawing up of medication) by a parent |
| Healthcare provider medication administration | Relates to the process of giving medication, e.g., route / rate (as opposed to the dosing and drawing up of medication) by a HCP |
| Intervention sensitivity / specificity | Relates to the sensitivity / specificity outcome measure of the tested intervention |
| Intervention validity | Relating to the validity of the results generated as a byproduct of usage of the intervention. Normally against a standard. |
| Medical ‘error’ or incidents | Relates to interventions whereby the outcome measure looks at the rate of medical error over time, serious significant events, safety incidents, or discharge-related care failures. |
| Concordance | Relating to the measurement of the intervention against a previously existing professional standard |
| Healthcare provider knowledge | Relates to the knowledge and understanding of topics covered by then intervention, where knowledge is measured |
| Parental intervention use | Relates to whether or not parents access or use available interventions when given a choice |
| Length of stay | Relates to during of a patient inpatient stay on a ward, e.g., the Intensive Care Unit |
| Medical history completeness | Relates to the completeness, e.g., name, dose, frequency, of a medical history of a patient. |
| Communication rate | Relates to a documented / measured communication between HCP and a family member |
| Parental stress | Relates to measuring the level of stress experienced by a parent |
| Medication reconciliation errors | Relating to the medication reconciliation rate |
| Prototype development | Relates to the design, development, and evaluation a prototype for the intervention in question |
| Prescription ‘error’ | Errors or mistakes relating to orders or prescriptions written for patients |
| Seeking repeated medical review | Relates to patients requiring a re-admission to hospital, attending an emergency department, or re-consulting a doctor |
| Work or school days missed | Related to number of days missed at work (parent) or school (child) due to the condition under investigation |
| Timely access to medication | Relates to parents accessing medication after hospital discharge |
| Clinical safety | Relating to measures as described by the authors, for example, malaria identification. |

| Author and Year | Location / Setting | Study design | Abstract / Full text | Key safety area | Age of children | Intervention category | Outcome measure | Reported effectiveness | Level | Continuum | IAP2 level |
| --- | --- | --- | --- | --- | --- | --- | --- | --- | --- | --- | --- |
| Yust *et al.* 2012 (1) | Secondary  Care | Interventional | Abstract | Medication | Under 6 | Educational intervention (parent) | Parental knowledge  Parental perception | Post intervention caregivers gave a lower safety score for CCM. Post education score 2.14 with a difference of 2.4 points (95%CI 2.1, 2.8, p<0.00001). | Direct Care | Consultation | Inform |
| Yubonpunt *et al.* 2021 (2) | Secondary  Care | Quasi  Experimental | Full-Text | Medication  Communication  Administration | 3-7 years | Multi-component  Written information or instructions  Audio or visual information | Parental knowledge | Intervention group had a higher knowledge score (mean difference=4.33, P<0.001) and total scores for engagement (mean difference=0.73, P <0.001). | Direct Care | Involvement | Inform |
| Yin *et al.* 2017a (3) | Secondary  Care | RCT | Full-Text | Medication | ≤8 years | Audio or visual information | Parental dosing accuracy | We found that pictograms were associated with statistically significant reductions in large overdosing errors, with a trend for reduction in any error. | Direct Care | Consultation | Inform |
| Yin *et al.* 2011(4) | Secondary  Care | Experimental | Full-Text | Medication | Not Specified | Audio or visual information | Parental dosing accuracy | Text-plus-pictogram recipients were less likely to make an error compared to text-only recipients (43.9% vs 59.0%, P= .01; absolute risk reduction, 15.2% [95% confidence interval, 3.8–26.0]; number needed to treat, 7 [4–26]). | Direct Care | Consultation | Inform |
| Yin *et al.* 2017b (5) | Secondary  Care | RCT | Full-Text | Medication  Diagnosis | 2-12 years | Written information or instructions | Parental knowledge  Parental perception | Parents who received the low-literacy plan were: 1) less likely to make an error in knowledge of medications to take everyday and when sick compared to parents who received the standard plan (63.0 vs. 77.3%, p = 0.03; adjusted odds ratio [AOR] = 0.5 [95% confidence interval: 0.2–0.9]), 2) less likely to make an error regarding spacer use (14.0 vs. 51.1%, p < 0.001; AOR = 0.1 [0.06–0.3]).  Parents found the low-literacy pictogram and photograph-based action plan to be easier to understand (p = 0.07). | Direct Care | Consultation | Consult |
| Yin *et al.* 2008 (6) | Secondary  Care | RCT | Full-Text | Medication | 30 days to 8 years | Communication techniques / counselling strategies  Audio or visual information | Parental knowledge  Dosing accuracy  Medication adherence | Intervention caregivers had fewer errors in observed dosing accuracy (20% deviation from prescribed dose) compared with caregivers who received routine counselling (daily dose: 5.4% vs 47.8%; absolute risk reduction [ARR],42.4% [95% confidence interval, 24.0%-57.0%]). | Direct Care | Consultation | Inform |
| Yin *et al.* 2014 (7) | Secondary  Care | Cross sectional | Full-Text | Medication | <9 years | Communication techniques / counselling strategies  Audio or visual information | Parental dosing accuracy | Advanced counselling and instrument provision in the ED were associated with decreased errors (30.5 vs 46.4%, P = .01; 21.8 vs 45.7%, P = .001). | Direct Care | Consultation | Inform |
| Weingart *et al.* 2013 (8) | Secondary  Care | QI | Full-Text | Communication  Documentation | Not Specified | Standardising a process/framework | Non-parental  Healthcare provider perception | Baseline measures of overall handoff score (OHS) showed no improvement. Further modifications were made to both education and process with an improved OHS (88.8±11.1, P<0.05). | Direct Care | Involvement | Involve |
| Wei *et al* 2017 (9) | Primary  Care | RCT | Full-Text | Medication | 2-14 years | Multi-component  Educational intervention (healthcare provider)  Educational intervention (parent)  Audio or visual information  Written information or instructions | Non-parental  Prescription rates | The antibiotic prescription rate at the individual level decreased from 82% (1936/2349) to 40% (943/2351) in the experimental group, and from 75% (1922/2548) to 70% (1782/2552) in the control group. Absolute risk reduction in the antibiotic prescribing rate of 29% (95% CI –42 to –16). | Direct Care | Consultation | Inform |
| Webster *et al* 2015 (10) | Secondary  Care | QI | Abstract | Communication  Diagnosis | Not Specified | Standardising a process/framework  Family-activated escalation of care  Audio or visual information | Parental awareness of intervention  Staff awareness of intervention  Healthcare provider adherence to intervention | Correct documentation.  No parental concerns.  Appropriate case management. | Direct Care | Involvement | Involve |
| Walsh *et al* 2013 (11) | Secondary  Care | Interventional | Full-Text | Medication | 0-16 years | Proactive risk assessment | Parent-generated safety strategies | Parents developed a 13-step process map, with two to 19 failure modes per step.  Parents identified 69 failure modes that could occur in the process of home use of oral chemotherapy after a change in medication dose. | Organisational Design | Involvement | Collaborate |
| Walsh *et al* 2014 (12) | Secondary  Care | QI | Full-Text | Medication  Communication | Mean age 7.4 years | Web-based | Intervention feasibility  Parental perception | Overall, the rate of errors was 4.7 per 100 medications before and 4.4 per 100 medications after the intervention.  High ratings for acceptability and usefulness. | Direct Care | Involvement | Collaborate |
| Verzantvoort *et al* 2018 (13) | Primary  Care | Cross sectional | Full-Text | Diagnosis | Child and adult | Mobile application | Parental perception | Need to see a doctor: a sensitivity of 84% and a specificity of 74%. The app was rated as ‘neutral’ to ‘very clear’ by 87%, and 89% were ‘neutral’ to ‘very satisfied’. | Direct Care | Consultation | Inform |
| Uong *et al.* 2021 (14) | Secondary  Care | QI | Full-Text | Communication | 0-17 years | Multi-component  Communication techniques / counselling strategies  Audio or visual information  Written information or instructions | Parental knowledge | Increase mean correct response rate from 37% to 62%. No difference in readmission rates. | Direct Care | Involvement | Involve |
| Trahan *et al.* 2022 (15) | Secondary  Care | Pre/Post Interventional | Full-Text | Communication | Not Specified | Standardising a process/framework | Healthcare provider adherence to intervention  Parental perception | The Rounds Quality Score was significantly improved after checklist implementation, the pre- and post-intervention scores being 8.24 and 9.61/10, respectively (P-value <0.001).  For parent questions , pre- and post-satisfaction p=0.228, 90.5 to 92.3 (not a significant difference). Patient and parent satisfaction were rated higher with the use of the checklist in most areas, e.g., 'felt included' (p=0.002). | Direct Care | Consultation | Consult |
| Thakur *et al* 2021 (16) | Secondary  Care | Pre/Post Interventional | Full-Text | Medication  Communication | 10 Months – 8 years | Interviewing parents or asking specific questions  Audio or visual information | Parental knowledge  Parental perception | Caregivers in the post-intervention phase reported an increase in knowledge about opioid risks and safety, and were very satisfied with the consultation. | Organisational Design | Involvement | Involve |
| Stratton, C. and Powers, H. 2010 (17) | Secondary  Care | QI | Abstract | Communication  Documentation | Not Specified | Standardising a process/framework | Non-parental  Healthcare provider perception | "Positive findings", only 14.3% staff disagreeing it should be implemented. Reports improved patient safety and communication and interception of errors and dialogue between nurses and families. | Direct Care | Involvement | Inform |
| Smith, M. Y. and Tran, T. 2012 (18) | Not stated | Design / Development | Abstract | Medication | Not Specified | Web-based | Parental perception | Respondents preferred animation to traditional text-based interventions. | Direct Care | Consultation | Consult |
| Skeens *et al* 2022 (19) | Secondary  Care | Feasibility | Abstract | Medication | 8-17 years | Mobile application | Intervention feasibility  Parental perception | Caregivers identified information sharing, medication and symptom tracking, and reminders as important features. The mean system usability scale score among HCP was μ=84.2, which exceeded the standardized usability score of 68. | Direct Care | Consultation | Consult |
| Schumacher et al *2018 (20)* | Secondary /Community  Care | Prospective | Full-Text | Medication | Not Specified | Educational intervention (parent) | Parent medication administration | The number of processes with at least one handling error was reduced from 96.4% to 56.7% in rectal tube administration and from 66.7% to 13.5% in buccal administration (both p < 0.001). | Direct Care | Consultation | Inform |
| Schopf *et al* 2020 (21) | Secondary  Care | QI | Full-Text | Communication  Documentation | 4-64 years Average 17.6 years | Families actively present (+/- participate) | Non-parental  Healthcare provider perception  Healthcare provider adherence to intervention | In the psychiatric ED, nurse specialists were able to obtain more information (patient-specific data) from family / caregivers compared with the inpatient unit. There was a significant (P≤0.05) relationship between the source of the information and the location where it was obtained. | Direct Care | Consultation | Inform |
| Samuels-Kalow *et al.* 2024 (22) | Secondary  Care | Time series | Full-Text | Medication | 90 days – 11.9 years | Multi-component  Communication techniques / counselling strategies  Written information or instructions  Audio or visual information  Educational intervention (healthcare provider) | Parental dosing accuracy | After adjustment for language and health literacy, the adjusted odds ratio for error during the combined intervention phases was 0.52 (95% CI, 0.28-0.97) compared with baseline. | Direct Care | Consultation | Inform |
| Salas *et al* 2023 (23) | Secondary  Care | QI | Abstract | Medication | Mean age 11 | Multi-component  Written information or instructions  Audio or visual information  Interviewing parents or asking specific questions" | Parent medication administration | Since its implementation, no administrative errors have occurred. | Direct Care | Consultation | Inform |
| Riccioni *et al* 2021 (24) | Secondary  Care | QI | Abstract | Communication | Not Specified | Standardising a process/framework | Parental perception | Increase from 3.1 to 3.4 in "FCC" scores (family centred care). | Direct Care | Involvement | Consult |
| Price *et al* 2013 (25) | Secondary  Care | Feasibility | Full-Text | Diagnosis | <18 years | Web-based | Parental perception  Intervention sensitivity/specificity | Overall sensitivity of 93.3%, overall specificity of 12.9%. 91.4% described site as very easy to use.  Ninety percent of participants reported that the website was “very easy” to understand and use. | Direct Care | Consultation | Inform |
| Porter *et al* 2005 (26) | Secondary  Care | Cohort | Full-Text | Documentation  Medication  Communication | 1-12 years | Electronic interface | Intervention validity | The validity of medication name reported by the parents using the kiosk was 81% (95% CI 72%–87%) compared with the validity of physician’s documentation of medication names at 68%. | Direct Care | Consultation | Inform |
| Paydar-Darian *et al* 2022 (27) | Secondary  Care | QI | Full-Text | Communication | Not Specified | Standardising a process/framework  Educational intervention (parent) | Medical error or incident  Parental perception | No preventable SSEs in study period, compared with 4 in previous 2 years.  No change in family report of quality of information; sustained at 62%. | Direct Care | Consultation | Consult |
| Patidar *et al* 2021 (28) | Secondary  Care | Cross Sectional | Full-Text | Medication | Not Specified | Audio or visual information | Parental dosing accuracy | The prevalence of LMDE (liquid medicine drug errors) was lower in the OPMS group for each method of administration. The effect size (d Cohen) of the intervention OPMS was 1.86–6.4. Pictogram interventions reduced dosing errors by 48–54% points and improved mothers’ understanding of dosing. | Direct Care | Consultation | Inform |
| Park *et al* 2014 (29) | Secondary  Care | Cross Sectional | Full-Text | Diagnosis  Medication  Communication | Not Specified | Web-based | Parental knowledge | The total score of the caregivers’ awareness of patient safety had increased significantly after web-based self-learning patient safety education (t = 8.51, p = 0.001). | Direct Care | Involvement | Consult |
| Oliveira *et al* 2011 (30) | Secondary  Care | Qualitative | Abstract | Communication | Not Specified | Families actively present (+/- participate) | Parental perception | Parents’ report Family Centred Rounds enhance parent satisfaction. | Direct Care | Consultation | Consult |
| O'Connor *et al* 2017 (31) | Secondary  Care | RCT | Full-Text | Diagnosis | <18 years | Educational intervention (parent) | Concordance (intervention vs standard)  Parental perception | Concordance between photograph-based vs in-person diagnosis was 83% (95% CI, 71%-94%; κ = 0.81). Providing instructions significantly increased the quality of the best image submitted by each parent (mean PQRS score with instructions, 9.5; mean score without instructions, 8.9; P = .04). | Direct Care | Consultation | Inform |
| Naureckas *et al* 2020 (32) | Secondary  Care | RCT | Full-Text | Medication | 90 days – 11.9 years | Multi-component  Educational intervention (parent)  Written information or instructions  Communication techniques / counselling strategies | Parental dosing accuracy | There was a 58% increase in reporting safe dosing in the intervention group compared with the control group (relative risk 1.58; 95% CI 1.12 to 2.24). After adjustment for health literacy and language (adjusted relative risk 1.50; 95% CI 1.06 to 2.13). | Direct Care | Consultation | Consult |
| Mora Capín *et al* 2024 (33) | Secondary  Care | QI | Full-Text | Communication | Not Specified | Standardising a process/framework  Families actively present (+/- participate) | Parental perception | Caregivers’ perception regarding professionals’ identification of those who had attended to them improved (presentation included name and work position: 49.0% vs. 84.3%, p < 0.01). | Direct Care | Involvement | Inform |
| Lemiengre *et al* 2018 (34) | Primary  Care | RCT | Full-Text | Diagnosis  Communication  Medication | 1 month to 16 years | Multi-component  Interviewing parents or asking specific questions  Written information or instructions | Prescription rates | In comparison with usual care, POC CRP did not influence antibiotic prescribing, (adjusted odds ratio [AOR] 1.01, 95% confidence interval [CI] = 0.57 to 1.79). BISNA increased antibiotic prescribing (AOR 2.04, 95% CI = 1.19 to 3.50). In combination with POC CRP, this increase disappeared. | Direct Care | Consultation | Inform |
| Kosack *et al* 2022 (35) | Secondary  Care | Mixed Methods | Full-Text | Communication | Not Specified | Electronic interface | Parental perception  Healthcare provider perception | Spanish-speaking families exhibited statistically significant improvements in Child HCAHPS top box scores compared to English-speaking families (P = .001). Qualitative themes included a perceived reduction of communication errors and increased family participation. | Direct Care | Involvement | Inform |
| Kornburger *et al* 2013 (36) | Secondary  Care | QI | Full-Text | Medication  Communication | Not Specified | Educational intervention (healthcare provider)  Communication techniques / counselling strategies | Non-parental  Healthcare provider knowledge | Nursing staff demonstrated increased knowledge regarding health literacy and teach-back. | Direct Care | Consultation | Inform |
| Khan *et al* 2018 (37) | Secondary  Care | Pre/Post Interventional | Full-Text | Communication | <18 years | Multi-component  Standardising a process/framework  Educational intervention (healthcare provider)  Written information or instructions  Communication techniques / counselling strategies | Medical error or incident  Parental perception | The overall rate of medical errors (per 1000 patient days) was unchanged (41.2 (95% confidence interval 31.2 to 54.5) pre-intervention v 35.8 (26.9 to 47.7) post-intervention, P=0.21). Harmful errors (preventable adverse events) decreased by 37.9% (20.7 (15.3 to 28.1) v 12.9 (8.9 to 18.6), P=0.01) post-intervention. Families expressing concerns at the start of rounds and reading back plans increased. | Direct Care | Involvement | Collaborate |
| Kelly *et al* 2019 (38) | Secondary  Care | QI | Full-Text | Communication | Not Specified | Standardising a process/framework | Non-parental  Healthcare provider adherence to intervention | Staff performed 82% of checklist items. | Direct Care | Consultation | Involve |
| Kelly *et al* 2017 (39) | Secondary  Care | Cross Sectional | Full-Text | Communication  Administration  Medication | <12 years | Electronic interface | Parental perception  Parental intervention use | Overall, parent survey respondents were satisfied with the portal. The majority of parents accessed all functions. | Direct Care | Involvement | Involve |
| Hixson *et al* 2010 (40) | Secondary  Care | Prospective | Full-Text | Medication | 1-13 years | Audio or visual information | Parental dosing accuracy  Parental perception | Use of the Parental Analgesia Slide resulted in a reduction in the absolute percentage dose error from a median of 33.3 to 0% (P < 0.001) and an increase in the number of correct dosage intervals and frequencies (59 ⁄ 80 to 70 ⁄ 80, P = 0.046). Parents reported high levels of satisfaction. | Direct Care | Consultation | Inform |
| Hemmelgarn *et al* 2015 (41) | Secondary  Care | QI | Abstract | Medication  Communication | Not Specified | Multi-component  Interviewing parents or asking specific questions  Educational intervention (healthcare provider)  Standardising a process/framework | Parental perception | All families surveyed stated transitional care and administering home medications during transitional care improved their confidence about discharge and caring for their child at home. | Direct Care | Consultation | Consult |
| Graci 2013 (42) | Secondary  Care | QI | Full-Text | Communication | Neonatal | New ward round | Parental perception | Comfort rounds provide the NICU with a structured systematic approach to encourage communication with parents, assess a patient’s comfort, and evaluate the bedside environment. | Direct Care | Consultation | Inform |
| Goggin *et al* 2022 (43) | Primary  Care | RCT | Full-Text | Communication  Medication | 1-5 years | Multi-component  Educational intervention (parent)  Audio or visual information  Written information or instructions | Prescription rates  Parental perception | The odds of receiving inappropriate antibiotic treatment did not significantly vary by group (AOR 0.99, 95%CI: 0.52 to 1.89, p=0.98). | Direct Care | Involvement | Collaborate |
| Gardner *et al* 2022 (44) | Secondary  Care | QI | Full-Text | Communication | Not Specified | Standardising a process/framework | Healthcare provider satisfaction  Medical error or incident  Length of stay | Improved rating of rounding by nurses. Improved compliance with initiative throughout PDSA cycles.  Reduced number of safety interventions. | Direct Care | Consultation | Inform |
| Desnoyer *et al* 2012 (45) | Secondary  Care | Pilot Study | Abstract | Medication | <18 years | Interviewing parents or asking specific questions | Primary outcome not stated/not measurable | 34 Pharmaceutical Interventions were considered to have moderate to major usefulness. | Direct Care | Consultation | Inform |
| Dersch-Mills *et al* 2011 (46) | Secondary  Care | Observational | Full-Text | Medication | <18 years | Interviewing parents or asking specific questions | Non-parental  Medical history completeness | 51% medications recorded fully in admission history vs median completeness score of 100% in informed interview (interquartile range [IQR] 90% to 100%). | Direct Care | Consultation | Inform |
| Czulada *et al* 2016 (47) | Secondary  Care | QI | Full-Text | Communication | Not Specified | Multi-component  Family advisor on improvement team  Physical communication tool | Communication rate | Families who had documented communication from a doctor increased from 13% to 65%, P<0.001. | Organisational Design | Involvement | Collaborate |
| Cullen *et al* 2022 (48) | Primary  Care | QI | Full-Text | Medication | <21 years | Multi-component  Audio or visual information  Educational intervention (healthcare provider)  Communication techniques / counselling strategies  Written information or instructions | Parental knowledge | Overall understanding of medication use improved from 39.8% to 74%. Knowledge of accurate dosage improved from 50.9% to 76.8%. Correlation between syringe provision and caregiver understanding was strong (R 5 .84). | Direct Care | Consultation | Inform |
| Cox *et al* 2017 (49) | Secondary  Care | RCT | Full-Text | Communication | 0-17 years | Standardising a process/framework | Healthcare provider adherence to intervention  Parental perception | The intervention significantly increased the number of FCR checklist elements performed (β = 1.2, P < .001). Intervention families’ engagement and reports of safety climate were no different from usual care.  The intervention significantly increased the likelihood that families were asked for questions. | Direct Care | Involvement | Involve |
| Cody *et al* 2020 (50) | Secondary  Care | QI | Abstract | Medication  Communication  Administration | Not Specified | Standardising a process/framework | Primary outcome not stated/not measurable | Decreased medication errors, improved compliance with medication, effective communication between the team and the patients/caregivers. | Direct Care | Consultation | Inform |
| Chan *et al* 2017 (51) | Secondary  Care | RCT | Full-Text | Medication | 1 month – 8 years | Audio or visual information | Parental dosing accuracy | The intervention group made significantly fewer errors than did the control group (unadjusted OR: 0.192 (95% CI: 0.037, 0.990); P = 0.043). | Direct Care | Consultation | Inform |
| Carroll *et al* 2024 (52) | Secondary  Care | RCT | Full-Text | Medication  Communication | ≤6 years | Multi-component  Audio or visual information  Communication techniques / counselling strategies  Written information or instructions | Parental dosing accuracy  Parental knowledge | 41.7% had dosing errors, with significantly more errors in the standard counselling group compared with the intervention group (39 of 72 [54.2%] vs 24 of 79 [30.4%]; P = .003). | Direct Care | Consultation | Consult |
| Bertsche *et al* 2010 (53) | Secondary  Care | Prospective | Full-Text | Medication | Not Specified | Multi-component  Educational intervention (parent)  Educational intervention (healthcare provider)  Written information or instructions | Parent medication administration  Healthcare provider medication administration | The intervention shifted administration from nurses to parents (p<0.025). Errors were reduced by 32.5% in nurses and by 91.0% in parents (each p<0.001). | Direct Care | Consultation | Inform |
| Berg *et al* 2017 (54) | Secondary  Care | QI | Abstract | Communication | Not Specified | Physical communication tool  Educational intervention (parent) | Primary outcome not stated/not measurable | Not stated. Plans for future work via PDSA cycles. | Direct Care | Consultation | Consult |
| Anand *et al* 2015 (55) | Secondary  Care | RCT | Abstract | Medication  Communication | Not Specified | Families actively present (+/- participate) | Parental stress | There was significantly greater reduction in trait (p=0.004, state (p=0.01) and total anxiety (p=0.0012) pre and post transfer in the intervention group vs the control group. There were no differences in minor medication errors (36 vs 33). | Direct Care | Consultation | Inform |
| Abraham *et al* 2024 (56) | Community  Care | Qualitative | Full-Text | Medication  Communication | 12-18 years | Web-based | Parental perception | Parents have positive impressions of using the Family Medication Safety Plan. | Direct Care | Consultation | Involve |
| Trivedi *et al* 2020 (57) | Secondary  Care | QI | Full-Text | Medication | Not Specified | Multi-component  Audio or visual information  Educational intervention (healthcare provider)  Interviewing parents or asking specific questions | Non-parental  Medication reconciliation errors | An improvement in medicines reconciliation was seen from a mean of 69% in PDSA cycle 2 to 95% in PSDA cycle 3. | Direct Care | Consultation | Consult |
| Slagle *et al* 2010 (58) | Both | Design/ Development | Full-Text | Medication  Communication | 6-12 years | Web-based | Non-parental  Prototype development | We have completed the development and demonstration of a working prototype that allows a user to create a medication list, schedule medication doses, send alerts to a cell phone, and receive administration events. | Direct Care | Consultation | Involve |
| Sebastian *et al* 2024 (59) | Secondary  Care | Qualitative | Full-Text | Medication | Not Specified | Educational intervention (parent) | Parental knowledge | Caregiver-perceived understanding of medication indication for the intervention and control group was 96.37% and 93.18%, respectively (P = 0.007). | Direct Care | Consultation | Inform |
| Porter *et al* 2008 (60) | Secondary  Care | Quasi experimental | Full-Text | Medication  Communication | 3 months – 12 years | Electronic interface | Non-parental  Prescription error | Overall, ParentLink demonstrated no significant impact on the rate of error. | Direct Care | Consultation | Inform |
| Lake *et al* 2019 (61) | Secondary  Care | QI | Full-Text | Medication | Not Specified | Interviewing parents or asking specific questions | Medication reconciliation errors | 74 discrepancies were found, the most frequent being omission. | Direct Care | Consultation | Inform |
| Jolliff *et al* 2024 (62) | Community  Care | Design/ Development | Full-Text | Medication | Not Specified | Mobile application | Parental perception | Analyses yielded 11 challenges to medication safety and 11 corresponding design requirements that fit into three broader challenges. | Direct Care | Involvement | Collaborate |
| Huth *et al* 2023 (63) | Secondary  Care | Prospective | Full-Text | Communication  Administration | Not Specified | Multi-component  Standardising a process/framework  Interviewing parents or asking specific questions | Medical error or incident  Seeking repeated medical review | 36.4% decrease in the rate of incidents per discharge.  The 30-day ED visit rate was significantly lower after intervention (12.6% versus 3.4%, per 100 discharges, P = 0.05). | Direct Care | Consultation | Involve |
| Francis *et al 2009* (64) | Primary  Care | RCT | Full-Text | Medication  Communication | 6 months – 14 years | Written information or instructions | Seeking repeated medical review | Children in the intervention group were significantly less likely to receive a prescription for antibiotics at the index consultation. There were no significant differences in terms of parent measures. | Direct Care | Involvement | Consult |
| Marino et al 2023 (65) | Secondary  Care | QI | Full-Text | Communication | Not Specified | Physical communication tool | Parental perception | Acceptable to parents and feasible to use in clinical practice. | Direct Care | Involvement | Collaborate |
| Frush et al 2004 (66) | Secondary  Care | RCT | Full-Text | Medication | 3 months – 12 years | Written information or instructions | Parental dosing accuracy | The average deviation was higher for the group using conventional methods compared with the group using the color-coded method (25.8% vs 1.7%). The conventional group also had a higher percentage of participants overdosed (30% vs 6%). | Direct Care | Consultation | Inform |
| Hu *et al* 2013 (67) | Secondary  Care | Interventional | Full-Text | Medication | Not Specified | Multi-component  Audio or visual information  Educational intervention (parent) | Parental knowledge | The proportions of the caregivers in Groups 1, 2 and 3 who answered the questionnaire perfectly with 100% accuracy were 2%, 14% and 74%, respectively (p < 0.001). | Direct Care | Consultation | Inform |
| Heath *et al 2016 (68)* | Secondary  Care | QI | Full-Text | Communication | Not Specified | Multi-component  Educational intervention (healthcare provider)  Written information or instructions | Parental perception | Increased confidence in terms of raising concerns and having them listened to. | Direct Care | Involvement | Involve |
| Wirral Community Health and Care NHS Foundation Trust 2021 (69) | Primary/ Community  Care | QI | Non-academic publication | Administration  Communication  Documentation | Not Specified | Standardising a process/framework | Primary outcome not stated/not measurable | Report quality improvement efficiencies and roll out to include other services. | Organisational Design | Involvement | Involve |
| Seigel *et al* 2014 (70) | Secondary  Care | QI | Full-Text | Communication | Not Specified | Standardising a process/framework | Healthcare provider adherence to intervention  Healthcare provider perception  Communication rate | Improved staff satisfaction with rounds (81%). | Direct Care | Consultation | Inform |
| Ray *et al* 2009 (71) | Secondary  Care | QI | Full-Text | Communication | Not Specified | Multi-component  Family-activated escalation of care  Educational intervention (parent)  Written information or instructions | Parental knowledge  Parental intervention use | Family concern was noted as a reason for activation in 5% of all RRT calls.  Only 27% of families understand when and how to activate the RRT. | Direct Care | Involvement | Consult |
| Aini *et al* 2017 (72) | Secondary  Care | Quasi Experimental | Full-Text | Communication | Not Specified | Educational intervention (parent) | Parental knowledge | The result of the paired T-test showed a p-value <0,001 which means that there was a significant influence by way of education on parent’s speak up knowledge regarding patient safety (but not in all areas covered). | Direct Care | Consultation | Inform |
| Auger *et al* 2018 (73) | Secondary  Care | RCT | Full-Text | Communication | <18 years | Interviewing parents or asking specific questions | Seeking repeated medical review  Parental knowledge | Children in the intervention and control arms had similar reutilization rates for 30-day urgent health care services (intervention group, 77 [15.9%]; control group, 63 [13.1%]; P = .21).  Parents of children in the intervention group recalled more clinical warning signs at 14 days (mean, 1.8 [95% CI, 1.7-2.0] in the intervention group; 1.5 [95% CI, 1.4-1.6] in the control group. | Direct Care | Involvement | Inform |
| Cameron *et al* 2009 (74) | Secondary  Care | Observational | Full-Text | Communication | Not Specified | Families actively present (+/- participate) | Parental perception  Healthcare provider knowledge  Healthcare provider perception | Increased their overall satisfaction with their child’s care. In 57% of rounding events, at least 1 HCP learned new, pertinent information from the parents. | Direct Care | Consultation | Inform |
| Fiks *et al* 2015 (75) | Primary  Care | RCT | Full-Text | Communication  Administration  Documentation | 6-12 years | Electronic interface | Intervention feasibility  Parental perception  Work or school days missed | Parents reported that use improved their communication with the office, ability to manage asthma, and awareness of the importance of ongoing attention to treatment. Intervention parents missed fewer days of work due to asthma. | Direct Care | Partnership | Consult |
| Hueckel *et al* 2012 (76) | Secondary  Care | QI | Full-Text | Communication | Not Specified | Educational intervention (parent) | Parental knowledge  Parental intervention use | Families who received Condition H teaching ranged from 64% to 90% with a mean of 80%. | Direct Care | Involvement | Consult |
| Zimmer *et al* 2015 (77) | Secondary  Care | RCT | Full-Text | Medication | Not Specified | Written information or instructions  Educational intervention (parent) | Parental knowledge | Median knowledge deficits in the pharmaceutical counselling group were significantly lower than deficits in the control group, p=0.017. | Direct Care | Consultation | Inform |
| Kuo *et al 2012 (78)* | Secondary  Care | Cohort | Full-Text | Communication | <10 years | Standardising a process/framework | Parental perception | Compared with non-FCR (family centred rounds), FCR families were more likely to report consistent medical information (P < .001), the option of discussing care plan (P < .001), doctors listening carefully (P < .01), and doctors showing respect (P < .001). | Direct Care | Consultation | Inform |
| Ladak *et al* 2013(79) | Secondary  Care | Pre/Post Interventional | Full-Text | Communication | Not Specified | Standardising a process/framework | Parental perception  Healthcare provider perception  Length of stay | Parents’ ratings during the family-centred rounds were significantly higher for some parental satisfaction items, including decision making related to care (p = 0.01). No significant differences were found in health care professionals’ satisfaction between rounds. | Direct Care | Involvement | Inform |
| Latta *et al* 2008 (80) | Secondary  Care | QI | Full-Text | Communication | Not Specified | Families actively present (+/- participate) | Parental perception | Including parents on ward rounds at a  teaching hospital was viewed positively  by parents. | Direct Care | Involvement | Inform |
| Mallory *et al* 2017(81) | Secondary  Care | Time Series | Full-Text | Medication  Communication  Documentation | 0-18 years | Multi-component  Standardising a process/framework  Communication techniques / counselling strategies  Interviewing parents or asking specific questions | Parental knowledge  Seeking repeated medical review | Caregiver ability to teach-back essential home management information post discharge improved from 18% to 82%. | Direct Care | Consultation | Consult |
| Maor *et al* 2011 (82) | Primary  Care | Interventional | Full-Text | Medication | 8 Days – 16 years | Multi-component  Written information or instructions  Educational intervention (parent) | Parental perception | 10% increase in parents’ preferences to avoid antibiotic treatment when the necessity of treatment was in doubt and a 10% decrease in parents’ expectations for antibiotic treatment in parents who reported they noticed the intervention. | Direct Care | Consultation | Inform |
| McCann *et al* 2008 (83) | Secondary  Care | Quasi Experimental | Full-Text | Communication | Not Specified | Interviewing parents or asking specific questions | Non-parental  Healthcare provider perception | Nurses in the intervention group were significantly more likely to include parents in decision making (p=0.007); encourage parents to ask questions during their child’s hospital stay (p=0.005). | Direct Care | Involvement | Inform |
| Porter *et al 2000 (84)* | Secondary  Care | Validation | Full-Text | Documentation | <12 months | Electronic interface | Non-parental  Intervention validity | The validity of the electronic medical record interface data was high across the PMH elements (94% to 99%). | Direct Care | Consultation | Inform |
| Schnellinger *et al* 2010 (85) | Secondary  Care | RCT | Full-Text | Medication | 0-18 years | Educational intervention (parent) | Parental knowledge | Postintervention survey scores improved significantly in the pamphlet and video groups compared with baseline. | Direct Care | Consultation | Inform |
| Staveski *et al* 2016 (86) | Secondary  Care | Quasi Experimental | Full-Text | Diagnosis | 0-19 years | Multi-component  Educational intervention (healthcare provider)  Educational intervention (parent)  Written information or instructions | Parental knowledge | Surgical-site infections decreased from 27% in Cohort 1 to 2% in Cohort 2.  Parent knowledge scores increased from 1.76 ± 1.4 for Cohort 1 to 3.68 ± 0.852 for Cohort 2. | Direct Care | Consultation | Inform |
| Taylor *et al 2005 (87)* | Primary  Care | RCT | Full-Text | Medication | <24 months | Educational intervention (parent) | Non-parental  Prescription rates | There were no significant differences between children in the intervention and control groups for any of these outcomes (otitis media, sinusitis, and antibiotic prescriptions). | Direct Care | Consultation | Inform |
| Voirol *et al* 2018 (88) | Secondary  Care | RCT | Full-Text | Medication | Not Specified | Interviewing parents or asking specific questions | Parental knowledge  Timely access to medication | The intervention group was able to have obtained medications within 24 hours more often than the control group (84% vs 69%; p=0.027).  Caregivers’ knowledge of how to correctly administer medications did not differ between the 2 groups. | Direct Care | Consultation | Inform |
| Liang *et al* 2018 (89) | Secondary  Care | Descriptive | Full-Text | Communication  Documentation | Not Specified | Family engagement consultation service (peer to peer support) | Primary outcome note stated/not measurable | Development of a new variation of this engagement consultation service. | Direct Care | Involvement | Collaborate |
| Wu *et al* 2016 (90) | Secondary  Care | QI | Full-Text | Communication | Not Specified | Multi-component  Educational intervention (parent)  Written information or instructions | Medical error or incident  Parental perception  Seeking repeated medical review | Significant decrease in discharge-related care failures, from 34% in the first project quarter to 21% at the end of the collaborative (P < .05). Significant improvement in family perception of readiness for discharge, from 85% of families reporting the highest rating to 91% (P < .05). There was no improvement in unplanned 72-hour (0.7% vs 1.1%, P = .29) and slight worsening of the 30-day readmission rate (4.5% vs 6.3%, P = .05). | Direct Care | Consultation | Inform |
| Khan *et al* 2017 (91) | Secondary  Care | Prospective | Full-Text | Communication | 0-17 years | New ward round | Parental perception  Healthcare provider perception | Top-box parent experience improved for 1 of 4 domains: Experience and Communication with Nighttime Doctors (23.6% to 31.5%). Post-intervention, nurse-parent dyads demonstrated an increase in adjusted composite shared  understanding (36.2% pre-intervention to 48.2% post-intervention, p=.002). | Direct Care | Involvement | Collaborate |
| Finkelstein *et al* 2001 (92) | Primary  Care | RCT | Full-Text | Medication | 3 months – 6 years | Educational intervention (parent)  Written information or instructions | Non-parental  Prescription rates | Antibiotic dispensing for children 3 to <36 months old decreased by 0.41 antibiotics per person-year (18.6%) in intervention groups compared with 0.33 (11.5%) in control practices. | Direct Care | Consultation | Inform |
| Walsh *et al* 2021 (93) | Secondary  Care | RCT | Full-Text | Medication  Communication  Documentation | 4 months – 11 years | Electronic interface | Parent medication administration  Communication rate | Among children on prescription medications, PHP significantly increased discussion of medications at the physical, but not the number who brought medications to the visit; it had no impact on those not on prescription medications. | Direct Care | Consultation | Consult |
| Acker *et al* 2025 (94) | Secondary Care | RCT | Full-Text | Communication | 0-18 years | Web Based  Educational intervention (Parent) | Parental intervention use  Intervention feasibility | Among those randomized to the One-4-ALL group, 26 of 30 (87 %) successfully created an account and viewed the web-based educational content. | Direct Care | Consultation | Inform |
| Bravo *et al* 2024 (95) | Secondary Care | QI | Abstract | Communication  Documentation | Not stated | Educational intervention (Healthcare)  Standardising a process/framework | Healthcare provider adherence to intervention | Physician documentation of IS use or non-use in patients with NELP, from 10% to 76%. | Direct Care | Consultation | Inform |
| Carron *et al* 2024 (96) | Secondary Care | QI | Full-Text | Communication | 0-10 years | Audio or visual information  Educational intervention (parent) | Parental perception | Of all eligible encounters who responded to the survey, 69.7% of those shown a video favourably answered the target question, compared to 66.8% of those who were not shown a video. | Organisational Design | Consultation | Consult |
| Clement *et al* 2024 (97) | Primary Care | Qualitative | Full-Text | Communication  Medication | 0-9 years | Written information or instructions  Interviewing parents or asking specific questions  Standardising a process/framework | Healthcare provider perception  Prescription rate  Non-Parental | Most clinicians liked the intervention because it was quick and easy to use, it helped elicit carer concerns, and reassured clinicians and carers of the appropriateness of treatment decisions. | Direct Care | Involvement | Involve |
| De Pins *et al* 2024 (98) | Secondary Care | QI | Full-Text | Communication  Documentation | Not stated | Written information or instructions  Educational intervention (Healthcare)  Standardising a process/framework | Healthcare provider adherence to intervention | AVS patient instruction completion rates increased from a mean of 39.4% to 85.0%. Provider education reduced mean jargon usage in patient instructions, from 8.2 to 3.9 jargon terms. | Direct Care | Consultation | Consult |
| Gutierrez-Wu *et al* 2024 (99) | Secondary Care | Interventional | Full-Text | Communication | <13 years | Electronic interface  Written information or instructions | Parental intervention use  Parental perception | Most parents endorsed interest in HIT, however only 12% activated portals. Post-intervention, there were non-significant increases in using portals to make appointments, receive reminders, send/receive messages, and view labs/instructions. Uses of study tablets included video visits (56%), health/COVID information (16%), and schoolwork (16%). | Direct Care | Consultation | Inform |
| Hoyt-Austin *et al* 2025 (100) | Secondary Care | Feasibility | Full-Text | Communication | <365 days | New ward round | Parental intervention use  Intervention feasibility | Protocol paper ( no results). | Direct Care | Consultation | Inform |
| Shaikh *et al* 2024 (101) | Community | Development | Full-Text | Communication  Medication | Not stated | Standardising a process/framework  Communication techniques / counselling strategies  Written information or instructions  Audio or visual information | Primary outcome not stated/not measurable | 100% of participants responded that they were likely or very likely to adopt or implement strategies. | Direct Care | Consultation | Involve |
| Costa *et al* 2024 (102) | Secondary Care | Quasi-Experimental | Full-Text | Communication | Neonates | Educational intervention (Parents)  Communication tool | Parental perception  Parental knowledge | There is a considerable change in behaviour regarding the PS actions related to the different groups. There is a significant difference in relation to the change of attitude regarding the questions: “Do I ask the professionals to check the bracelet before care?”; “Do I remind the professional about the existence of any allergies?” and “Do I remind the professionals to confirm data before medication?”, in the Experimental Group. | Direct Care | Involvement | Inform |
| Topshee *et al* 2024 (103) | Primary Care | QI | Abstract | Administration | 3 months - 5 years | New service | Parental intervention use  Intervention feasibility | Almost two thirds (63.3%) of patients returned for a repeat visit with 132 total clinical encounters noted over a 9-month period (17 clinics total). Prescriptions for children comprising primarily antibiotics. | Direct Care | Consultation | Inform |
| Unger *et al* 2025 (104) | Community | RCT | Full-Text | Communication  Administration | Neonates | New service | Mortality | Text communication did not decrease neonatal mortality compared to standard of care. Most neonates died before discharge from the facility. | Direct Care | Consultation | Inform |
| Wener *et al* 2024 (105) | Community | RCT | Full-Text | Communication\  Medication | 0-16 years | Mobile application | Parent medication administration | The Meds@HOME mobile app provides a promising strategy for improving PCG medication safety for CMC who take high-risk medications. | Direct Care | Involvement | Involve |
| Pilarz *et al* 2025 (106) | Secondary Care | Quasi-Experimental | Abstract | Communication | Not Specified | Family activated care | Parental intervention use  Healthcare provider perception | Pre-intervention, there were an average of 1.01 interpreter encounters per patient-day, compared to a post-intervention average of 1.35 interpreter encounters per patient-day, which was not statistically significant (p = 0.22).  The total duration of interpreter use per patient-day increased from an average of 7.6 minutes pre-intervention to 16.1 minutes post-intervention (p = 0.02). On a five-point scale, the mean feasibility score was 4.5, acceptability was 4.7, and appropriateness was 4.7. | Direct Care | Consultation | Inform |
| Ahmed *et al* 2024 (107) | Secondary Care | QI | Abstract | Communication  Administration | Not stated | Standardising a process/framework  Education (Healthcare)  New service | Healthcare provider perception  Parental Perception  Parental intervention use | Three surveys were conducted. The parents’ survey confirmed two-thirds respondents (36, 60%) used OA-facility; their preferred communication mode was direct-line to ward phone (33, 55%), although parents supported additional virtual options (mobile or email). Staff surveys suggests no seasonal impact on call-activity (>5 calls/respondent: winter 47% vs summer 44%) but post-intervention-survey attitudes improved towards EPR documentation. | Direct Care | Consultation | Inform |
| Flaherty *et al* 2024 (108) | Primary Care | Cohort | Full-Text | Communication  Administration | <=10 years | Education (Healthcare)  New Service | Intervention feasibility  Intervention safety | Safety and feasibility thresholds were met. 98% of febrile cases received rapid diagnostic testing for malaria; 4% tested positive. At 10 days, 97% of cases were improving/well, and no severe adverse events were reported. The median lengths of the telemedicine assessment, time to delivery, and in-person assessments were 9, 49, and 43 min, respectively. 99% of participants expressed interest in using the service again. There was fair congruence between paired telemedicine and in-person assessments for vital sign assessments and mild/moderate triage decisions. | Direct Care | Consultation | Inform |
| Quinonez *et al* 2025 (109) | Secondary Care | Qualitative | Full-Text | Communication | Not stated | Families actively present (+/- participate) | Healthcare provider perception | Twenty provider interviews were conducted. Four categories emerged: (1) positive impact of FP, (2) negative impact of FP, (3) limitations to achieving FP, and (4) policies and procedures for FP. | Direct Care | Consultation | Inform |
| Madduri *et al* 2024 (110) | Secondary Care | QI | Full-Text | Communication | Not stated | Standardising a process/framework  Educational intervention (Healthcare) | Healthcare provider adherence to intervention | The mean documented FCR was 33% at baseline. The mean FCR increased to 81% with the implementation of the EHR-FCR checklist, and then decreased to 73% during the sustain period. A statistically higher proportion of families with LOE participated in FCR during the sustain period (87% vs 72%, P <.01). | Direct Care | Consultation | Inform |
| Beard *et al* 2025 (111) | Secondary Care | QI | Full-Text | Communication  Medication | Neonates | Educational intervention (Parents)  Standardising a process/framework | Seeking repeated medical review  Parental perception  Healthcare provider perception | Staff-assessed caregiver DC readiness scores were not assessed at baseline but met or exceeded a 90% mean score during intervention and maintenance phases. Compliance reached 100% after integration into the DC process. The mean DC class time in English was 1.5 hours and 2 hours with interpretation. DC class attendance, initially non-existent, reached 100% with DC navigator role initiation and was sustained through the study DCRFs were common, decreasing from 23% (79/342) at baseline to 11% (46/424) and 17% (114/687) in contacted caregivers during intervention and maintenance phases, respectively. Readmissions were infrequent with no differences between phases. | Direct Care | Consultation | Inform |

Supplementary File 5.

**References of articles included in scoping review**

1. Yust E, McKinney S, Slattery A, King W, Monroe K. Parental use of cold and cough medicine in children younger than six. Annals of Emergency Medicine. 2012;1:S26-S7.
2. Yubonpunt P, Kunno J, Viwattanakulvanid P, Rungsihirunrat K. Effect of multi-component program on promoting safety of hospitalized children. Evidence Based Care Journal. 2021;11(1):51-61.
3. Yin HS, Parker RM, Sanders LM, Mendelsohn A, Dreyer BP, Bailey SC, et al. Pictograms, Units and Dosing Tools, and Parent Medication Errors: A Randomized Study. Pediatrics. 2017;140(1).
4. Yin HS, Mendelsohn AL, Fierman A, Van Schaick L, Bazan IS, Dreyer BP. Use of a pictographic diagram to decrease parent dosing errors with infant acetaminophen: A health literacy perspective. Acad Pediatr. 2011;11(1):50-7.
5. Yin HS, Gupta RS, Mendelsohn AL, Dreyer B, van Schaick L, Brown CR, et al. Use of a low-literacy written action plan to improve parent understanding of pediatric asthma management: A randomized controlled study. Journal of Asthma. 2017;54(9):919-29.
6. Yin HS, Dreyer BP, van Schaick L, Foltin GL, Dinglas C, Mendelsohn AL. Randomized controlled trial of a pictogram-based intervention to reduce liquid medication dosing errors and improve adherence among caregivers of young children. Archives of Pediatrics & Adolescent Medicine. 2008;162(9):814-22.
7. Yin HS, Dreyer BP, Moreira HA, van Schaick L, Rodriguez L, Boettger S, et al. Liquid Medication Dosing Errors in Children: Role of Provider Counseling Strategies. Academic Pediatrics. 2014;14(3):262-70.
8. Weingart C, Herstich T, Baker P, Garrett ML, Bird M, Billock J, et al. Making good better: Implementing a standardized handoff in pediatric transport. Air Medical Journal. 2013;32(1):40-6.
9. Wei X, Zhang Z, Walley JD, Hicks JP, Zeng J, Deng S, et al. Effect of a training and educational intervention for physicians and caregivers on antibiotic prescribing for upper respiratory tract infections in children at primary care facilities in rural China: a cluster-randomised controlled trial. The Lancet Global Health. 2017;5(12):e1258-e67.
10. Webster MA, Haines C, Fraser J. The development and implementation of a policy promoting parental (patient) involvement in escalation of clinical care. Archives of Disease in Childhood. 2015;3:A235.
11. Walsh KE, Mazor KM, Roblin D, Biggins C, Wagner JL, Houlahan K, et al. Multisite parent-centered risk assessment to reduce pediatric oral chemotherapy errors. Journal of Oncology Practice. 2013;9(1):e1-e7.
12. Walsh KE, Biggins C, Blasko D, Christiansen SM, Fischer SH, Keuker C, et al. Home medication support for childhood cancer: Family-centered design and testing. Journal of Oncology Practice. 2014;10(6):373-7.
13. Verzantvoort NCM, Teunis T, Verheij TJM, van der Velden AW. Self-triage for acute primary care via a smartphone application: Practical, safe and efficient? Plos One. 2018;13(6).
14. Uong A, Philips K, Hametz P, Dunbar J, Jain P, O'Connor K, et al. SAFER Care: Improving Caregiver Comprehension of Discharge Instructions. Pediatrics. 2021;147(4).
15. Trahan C, Hui AY, Binepal N. Standardization of rounds on a general paediatric ward: Implementation of a checklist to improve efficiency, quality of rounds, and family satisfaction. Paediatr Child Health. 2022;27(2):111-7. doi:10.1093/pch/pxac003.
16. Thakur T, Chewning B, Zetes N, Lee JTY. Involving caregivers in design and assessment of opioid risk and safety communication intervention in children. Patient Education and Counseling. 2021;104(10):2432-6.
17. Stratton C, Powers H. Family centered care and the implementation of bedside report. Pediatric Blood and Cancer. 2010;55:981.
18. Smith MY, Tran T. Development of a new type of risk minimization tool to reduce medication errors. Pharmacoepidemiology and Drug Safety. 2012;3:349-50.
19. Skeens M, Ralph J, Sezgin E, Guttoo P, Vannatta K, Bajwa R, et al. Feasibility and Acceptability of an Mhealth App (Bmt4me) for Adherence and Symptom Tracking Using a Multidisciplinary and Family-Centered Approach in Pediatric Hematopoietic Stem Cell Transplant. Pediatric Blood and Cancer. 2022;69:S48.
20. Schumacher PM, Kaune A, Merkenschlager A, Bernhard MK, Kiess W, Neininger MP, et al. Optimizing parents' performance in anticonvulsant rescue medication administration. Epilepsy and Behavior. 2018;84:37-43.
21. Schopf D, Stark S, Chiappetta L, Mahmoud K, Fradkin D, Mitchell AM. An alternative admission process for patients with an autism spectrum disorder and/or an intellectual disability. Learning Disability Practice. 2020;23(1):26-32.
22. Samuels-Kalow ME, Tassone R, Manning W, Cash R, Davila-Parrilla L, Hayes BD, et al. Analysis of a Medication Safety Intervention in the Pediatric Emergency Department. JAMA Network Open. 2024;7(1):e2351629-e.
23. Salas NM, Barksdale T, Lee Y. Descriptive analysis of a pediatric anticoagulation stewardship initiative to improve anticoagulation safety: a quality improvement project. Res Pract Thromb Haemost. 2023;7(1):e100184. doi:10.1002/rth2.12758
24. Riccioni M, Gazzaneo MC, Sallans C, Trevino R, Bavare A. Improvement of family centered care during rapid response events. Pediatric Critical Care Medicine. 2021;22:114.
25. Price RA, Fagbuyi D, Harris R, Hanfling D, Place F, Taylor TB, et al. Feasibility of Web-Based Self-Triage by Parents of Children With Influenza-Like Illness - A Cautionary Tale. Jama Pediatrics. 2013;167(2):112-8.
26. Porter SC, Kohane IS, Goldmann DA. Parents as partners in obtaining the medication history. J Am Med Inform Assoc. 2005;12(3):299-305. doi:10.1197/jamia.M1713.
27. Paydar-Darian N, Stack AM, Volpe D, Gerling MJ, Seneski A, Eisenberg MA, et al. Improving Discharge Safety in a Pediatric Emergency Department. Pediatrics. 2022;150(5).
28. Patidar P, Mathur A, Pathak A. Can use of pictograms reduce liquid medication administration errors by mothers? An interventional study. BMC Psychology. 2021;9(1).
29. Park BK, Lee E. Effects of my child's safety web-based program for caregivers of children with cancer in South Korea. Healthcare Informatics Research. 2014;20(3):199-208.
30. Oliveira C, Mittal V, Lee B, Martin B, Patel R, Shetgiri R, et al. Do family-centered rounds (FCRS) improve parent satisfaction, communication, co-ordination of care, patient safety, outcomes, hand-offs, and trainees education? A qualitative study of parents of hospitalized children. Journal of Investigative Medicine. 2011;59:490-1.
31. O'Connor DM, Jew OS, Perman MJ, Castelo-Soccio LA, Winston FK, McMahon PJ. Diagnostic Accuracy of Pediatric Teledermatology Using Parent-Submitted Photographs: A Randomized Clinical Trial. JAMA Dermatology. 2017;153(12):1243-8.
32. Naureckas Li C, Camargo CA, Jr., Faridi M, Espinola JA, Hayes BD, Porter S, et al. Medication Education for Dosing Safety: A Randomized Controlled Trial. Annals of emergency medicine. 2020;76(5):637-45.
33. Mora Capín A, Jové Blanco A, Oujo Álamo E, Muñoz Cutillas A, Barrera Brito V, Vázquez López P. Involving the Patient and Family in the Transfer of Information at Shift Change in a Pediatric Emergency Department. Joint Commission Journal on Quality & Patient Safety. 2024;50(5):357-62.
34. Lemiengre MB, Verbakel JY, Colman R, De Burghgraeve T, Buntinx F, Aertgeerts B, et al. Reducing inappropriate antibiotic prescribing for children in primary care: a cluster randomised controlled trial of two interventions. Br J Gen Pract. 2018;68(668):e204-e10.
35. Kosack A, Carson SL, Bravo Montenegro EY, Owen M, Dodge S, Sim M, et al. Improving Patient Experience Scores Using Simultaneous Interpretation on Family-Centered Rounds. Hospital Pediatrics. 2022;12(12):1019-29.
36. Kornburger C, Gibson C, Sadowski S, Maletta K, Klingbeil C. Using "teach-back" to promote a safe transition from hospital to home: An evidence-based approach to improving the discharge process. Journal of Pediatric Nursing. 2013;28(3):282-91.
37. Khan A, Spector ND, Baird JD, Ashland M, Starmer AJ, Rosenbluth G, et al. Patient safety after implementation of a coproduced family centered communication programme: multicenter before and after intervention study. BMJ. 2018;363:k4764. doi:10.1136/bmj.k4764.
38. Kelly MM, Xie AA, Li YQ, Cartmill R, Cox ED, Brown RL, et al. System Factors Influencing the Use of a Family-Centered Rounds Checklist. Pediatric Quality & Safety. 2019;4(4).
39. Kelly MM, Hoonakker PL, Dean SM. Using an inpatient portal to engage families in pediatric hospital care. Journal of the American Medical Informatics Association. 2017;24(1):153-61.
40. Hixson R, Franke U, Mittal R, Hamilton M. Parental calculation of pediatric paracetamol dose: a randomized trial comparing the Parental Analgesia Slide with product information leaflets. Pediatric Anesthesia. 2010;20(7):612-9.
41. Hemmelgarn T, Carmichael J, Wieman R, Bucuvalas J. Improving the discharge process in pediatric liver/small bowel transplant patients through an interdisciplinary approach: Evolving improvement initiatives. Pediatric Transplantation. 2015;1:149.
42. Graci A. A rounding system to enhance patient, parent, and neonatal nurse interactions and promote patient safety. J Obstet Gynecol Neonatal Nurs. 2013;42(2):239-42. doi:10.1111/1552-6909.12011.
43. Goggin K, Hurley EA, Lee BR, Bradley-Ewing A, Bickford C, Pina K, et al. Let's Talk About Antibiotics: a randomised trial of two interventions to reduce antibiotic misuse. BMJ Open. 2022;12(11).
44. Gardner MK, Amado PJ, Baig MU, Mohiuddin S, Harden A, Ewing LJ, et al. Implementing Rounding Checklists in a Pediatric Oncologic Intensive Care Unit. Children-Basel. 2022;9(4).
45. Desnoyer A, Baruchel A, Brion F, Roupret-Serzec J. Development and implementation of a pharmaceutical consultation in a paediatric haematology unit: A pilot study. European Journal of Hospital Pharmacy: Science and Practice. 2012;19:252.
46. Dersch-Mills D, Hugel K, Nystrom M. Completeness of information sources used to prepare best possible medication histories for pediatric patients. Can J Hosp Pharm. 2011;64(1):10-5. doi:10.4212/cjhp.v64i1.979
47. Czulada L, Leino P, Willis TS. Partnering With a Family Advisor to Improve Communication in a Pediatric Intensive Care Unit. American Journal of Medical Quality. 2016;31(6):520-5.
48. Cullen SM, Osorio SN, Abramson EA, Kyvelos E. Improving Caregiver Understanding of Liquid Acetaminophen Administration at Primary Care Visits. Pediatrics. 2022;150(2).
49. Cox ED, Jacobsohn GC, Rajamanickam VP, Carayon P, Kelly MM, Wetterneck TB, et al. A Family-Centered Rounds Checklist, Family Engagement, and Patient Safety: A Randomized Trial. Pediatrics. 2017;139(5).
50. Cody SA, Ahmed-Winston S, Baldwin C. Planning for Success: A Multidisciplinary Approach to Complex Discharges in Pediatric Blood and Marrow Transplant. Biology of Blood and Marrow Transplantation. 2020;26:S383.
51. Chan HK, Aswad E, Ho YE. Influences of pictogram-based instructions in paediatric drug labelling on dosing accuracy among caregivers: a pilot study from Malaysia. Journal of Pharmaceutical Health Services Research. 2017;8(2):131-4.
52. Carroll AR, Johnson JA, Stassun JC, Greevy RA, Mixon AS, Williams DJ. Health literacy-informed communication to reduce discharge medication errors in hospitalized children: a randomized clinical trial. JAMA Netw Open. 2024;7(1):e2350969. doi:10.1001/jamanetworkopen.2023.50969.
53. Bertsche T, Bertsche A, Krieg EM, Kunz N, Bergmann K, Hanke G, et al. Prospective pilot intervention study to prevent medication errors in drugs administered to children by mouth or gastric tube: a programme for nurses, physicians and parents. Quality & Safety in Health Care. 2010;19(5).
54. Berg RBS, Stubbs MT, Dublon VE, Runnacles J. Championing parental concerns on a paediatric ward. Archives of Disease in Childhood. 2017;102:A100.
55. Anand V, Williams E, Elgendi M, Meakins L, Cunningham C, McCrady H, et al. Parental presence at cardiac intensive care unit bedside transfer rounds reduces parental anxiety: results of a randomized controlled trial. Circulation. 2015;132(Suppl 3):A135. Presented at: American Heart Association Scientific Sessions; 2015.
56. Abraham O, Klubertanz GC, Maday EN. Parents' perspectives on using a Family Medication Safety Plan designed to promote appropriate opioid prescription practices with adolescents. Journal of the American Pharmacists Association. 2024;64(3).
57. Trivedi A, Sharma S, Ajitsaria R, Davey NJ. Please reconcile, not wait a while. Arch Dis Child Educ Pract Ed. 2020;105(2):122-6. doi:10.1136/archdischild-2018-316356.
58. Slagle JM, Gordon JS, Harris CE, Davison CL, Culpepper DK, Scott P, et al. MyMediHealth - Designing a next generation system for child-centered medication management. Journal of Biomedical Informatics. 2010;43(5):S27-S31.
59. Sebastian S, Thornton H, Abdel-Rasoul M, Shaffery K. Patient-perceived understanding of home-going medication with transitions of care services at a pediatric institution. Journal of the American Pharmacists Association. 2024;64(3).
60. Porter SC, Kaushal R, Forbes PW, Goldmann D, Kalish LA. Impact of a patient-centered technology on medication errors during pediatric emergency care. Ambulatory Pediatrics. 2008;8(5):329-35.
61. Lake N, Nawer H, Wagner D. Piloting Medication Histories in a Pediatric Postanesthesia Care Unit. Journal of Perianesthesia Nursing. 2019;34(1):117-23.
62. Jolliff A, Coller RJ, Kearney H, Warner G, Feinstein JA, Chui MA, et al. An mHealth Design to Promote Medication Safety in Children with Medical Complexity. Applied Clinical Informatics. 2024;15(1):45-54.
63. Huth K, Hotz A, Emara N, Robertson B, Leaversuch M, Mercer AN, et al. Reduced Postdischarge Incidents After Implementation of a Hospital-to-Home Transition Intervention for Children With Medical Complexity. Journal of Patient Safety. 2023;19(7):493-500.
64. Francis N, Butler C, Hood K, Simpson S, Wood F, Nuttall J. Effect of using an interactive booklet about childhood respiratory tract infections in primary care consultations on reconsulting and antibiotic prescribing: a cluster randomised controlled trial. BMJ. 2009;339:b2885. doi:10.1136/bmj.b2885.
65. Marino L, Collaḉo N, Coyne S, Leppan M, Ridgeway S, Bharucha T, et al. The development of a communication tool to aid parent-centered communication between parents and healthcare professionals: a quality improvement project. Healthcare (Basel). 2023;11(20):2706. doi:10.3390/healthcare11202706.
66. Frush K, Luo X, P H, Higgins J. Evaluation of a method to reduce over-the-counter medication dosing error. Archives of pediatrics & adolescent medicine. 2004;158(7).
67. Hu H, Wu F-LL, Hu F-C, Yang H-Y, Lin S-W, Shen L-J. Effectiveness of Education Programs About Oral Antibiotic Suspensions in Pediatric Outpatient Services. Pediatrics & Neonatology. 2013;54(1):34-42.
68. Heath G, Montgomery H, Eyre C, Cummins C, Pattison H, Shaw R. Developing a Tool to Support Communication of Parental Concerns When a Child is in Hospital. Healthcare. 2016;4(1).
69. Wirral Community Health and Care NHS Foundation Trust. Working in partnership with families to develop a coordinated plan of care for their child. 2021. Available from: https://picker.org/wp-content/uploads/2022/01/Picker-NHS-Wirral-CS-Web.pdf
70. Seigel J, Whalen L, Burgess E, Joyner BL, Purdy A, Saunders R, et al. Successful Implementation of Standardized Multidisciplinary Bedside Rounds, Including Daily Goals, in a Pediatric ICU. The Joint Commission Journal on Quality and Patient Safety. 2014;40(2):83-AP2.
71. Ray E, Smith R, Massie S, Erickson J, Hanson C, Harris B, et al. Family Alert: Implementing Direct Family Activation of a Pediatric Rapid Response Team. Joint Commission journal on quality and patient safety / Joint Commission Resources. 2009;35:575-80.
72. Aini S, Mulatsih S, Lasmani P. The Effect of Education on Parents' "Speak Up" Knowledge Regarding Patients Safety in Hospital. Jurnal Ners. 2017;12:189-95.
73. Auger K, Shah S, Tubbs-Cooley H, Sucharew H, Gold J, Wade-Murphy S, et al. Effects of a 1-Time Nurse-Led Telephone Call After Pediatric Discharge: The H2O II Randomized Clinical Trial. JAMA pediatrics. 2018;172(9).
74. Cameron M, Schleien C, Morris M. Parental presence on pediatric intensive care unit rounds. The Journal of pediatrics. 2009;155(4).
75. Fiks A, Mayne S, Karavite D, Suh A, O'Hara R, Localio A, et al. Parent-reported outcomes of a shared decision-making portal in asthma: a practice-based RCT. Pediatrics. 2015;135(4).
76. Hueckel R, Mericle J, Frush K, Martin P, Champagne M. Implementation of condition help: family teaching and evaluation of family understanding. Journal of nursing care quality. 2012;27(2).
77. Zimmer J, Niemann D, Seltmann K, Fischer L, Christiansen H, Frontini R, et al. Managing of oral medicines in paediatric oncology: can a handbook and a pharmaceutical counselling intervention for patients and their parents prevent knowledge deficits? A pilot study. Eur J Hosp Pharm. 2024;23(2):100-5. doi:10.1136/ejhpharm-2015-000715.
78. Kuo D, Sisterhen L, Sigrest T, Biazo J, Aitken M, Smith C. Family experiences and pediatric health services use associated with family-centered rounds. Pediatrics. 2012;130(2).
79. Ladak L, Premji S, Amanullah M, Haque A, Ajani K, Siddiqui F. Family-centered rounds in Pakistani pediatric intensive care settings: non-randomized pre- and post-study design. International journal of nursing studies. 2013;50(6).
80. Latta LC, Dick R, Parry C, Tamura GS. Parental responses to involvement in rounds on a pediatric inpatient unit at a teaching hospital: a qualitative study. Acad Med. 2008;83(3):292-7. doi:10.1097/ACM.0b013e3181637e21.
81. Mallory L, Osorio S, Prato B, DiPace J, Schmutter L, Soung P, et al. Project IMPACT Pilot Report: Feasibility of Implementing a Hospital-to-Home Transition Bundle. Pediatrics. 2017;139(3).
82. Maor Y, Raz M, Rubinstein E, Derazne E, Ringel S, Roizin H, et al. Changing parents' opinions regarding antibiotic use in primary care. European journal of pediatrics. 2011;170(3).
83. McCann D, Young J, Watson K, Ware R, Pitcher A, Bundy R, et al. Effectiveness of a tool to improve role negotiation and communication between parents and nurses. Paediatric nursing. 2008;20(5).
84. Porter S, Silvia M, Fleisher G, Kohane I, Homer C, Mandl K. Parents as direct contributors to the medical record: validation of their electronic input. Annals of emergency medicine. 2000;35(4).
85. Schnellinger M, Finkelstein M, Thygeson M, Vander Velden H, Karpas A, Madhok M. Animated video vs pamphlet: comparing the success of educating parents about proper antibiotic use. Pediatrics. 2010;125(5).
86. Staveski S, Parveen V, Madathil S, Kools S, Franck L. Parent education discharge instruction program for care of children at home after cardiac surgery in Southern India. Cardiology in the young. 2016;26(6).
87. Taylor JA, Kwan-Gett TS, McMahon EM. Effectiveness of a parental educational intervention in reducing antibiotic use in children: a randomized controlled trial. Pediatr Infect Dis J. 2005;24(6):489-93. doi:10.1097/01.inf.0000164706.91337.5d.
88. Voirol P, Kayser SR, Chang CY, Chang QL, Youmans SL. Impact of pharmacists' interventions on the pediatric discharge medication process. The Annals of pharmacotherapy. 2004;38(10).
89. Liang WH, Madan-Swain A, Cronin RM, Jackson GP. Development of a Technology-Supported, Lay Peer-to-Peer Family Engagement Consultation Service in a Pediatric Hospital. AMIA Annu Symp Proc. 2018;2018:730-9.
90. Wu S, Tyler A, Logsdon T, Holmes NM, Balkian A, Brittan M, et al. A Quality Improvement Collaborative to Improve the Discharge Process for Hospitalized Children. Pediatrics. 2016;138(2).
91. Khan A, Baird J, Rogers J, Furtak S, Williams K, Allair B, et al. Parent and Provider Experience and Shared Understanding After a Family-Centered Nighttime Communication Intervention. Academic pediatrics. 2017;17(4):389-402.
92. Finkelstein JA, Davis RL, Dowell SF, Metlay JP, Soumerai SB, Rifas-Shiman SL, et al. Reducing antibiotic use in children: a randomized trial in 12 practices. Pediatrics. 2001;108(1):1-7. doi:10.1542/peds.108.1.1.
93. Walsh KE, Bacic J, Phillips BD, Adams WG. Misuse of Pediatric Medications and Parent-Physician Communication: An Interactive Voice Response Intervention. Journal of patient safety. 2021;17(3):e207-e13.
94. Acker SN, Meraz AI, Wilson SN, Mata C, Izere MF, Cooper E, et al. A Randomized Controlled Trial of Perioperative Education via e-health Technology to Ensure High Quality Equitable Care: One-4-ALL Initiative. Journal of Pediatric Surgery. 2025;60(4)
95. Bravo M, Paluck F, Taibah O, Amini A, Annan H, Tachejian S, et al. Improving language concordant care in the paediatric emergency department. Paediatr Child Health. 2024;29(Suppl 1):e16. doi:10.1093/pch/pxae067.033.
96. Carron HG, Valderrama G, Vukovic AA. A Quality Improvement Initiative Using Discharge Education Videos to Improve Communication with Families in a Pediatric Urgent Care. Patient Experience Journal. 2024;11(3):136-44.
97. Clement C, Ingram J, Blair PS, Cabral C, Hay AD, Seume P, et al. Implementation of the CHIldren with acute COugh (CHICO) intervention to improve antibiotics management: a qualitative study in primary care. Br J Gen Pract. 2024;74(743):e401-e7.
98. De Pins AM, Adu-Amankwah D, Shadman KA, Hess SM, Elaiho CR, Butler LR, et al. A Quality Improvement Project to Improve After-visit Summary Patient Instructions in a Pediatric Multidisciplinary Neuromuscular Program. Pediatric Quality and Safety. 2024;9(4):e743.
99. Gutierrez-Wu JC, McBride JP, Pittman A, Yang Y, Lin FC, Flower KB. Tech + touch: A pilot study to facilitate access to health information technology for Spanish-speaking parents. PEC Innovation. 2024;5.
100. Hoyt-Austin AE, Zerda EN, Tancredi DJ, Marcin JP, Ketchersid A, Horath ET, et al. Protocol for a single-arm feasibility trial of virtual family-centered rounds: increasing opportunities for family engagement among caregivers with language preference other than English. Pilot and Feasibility Studies. 2025;11(1).
101. Shaikh U, Kim JM, Yin SH. Implementing Strategies to Prevent Home Medication Administration Errors in Children With Medical Complexity. Clinical Pediatrics. 2024;63(7):877-81.
102. Costa MTTC, Ramalho MF, Diniz CG, Rocha PK, Santos LM, Corrêa AR, et al. Influence of a booklet about neonates’ safety on family members’ behavior and knowledge. Texto Contexto Enferm. 2024;33:e20230304. doi:10.1590/1980-265X-TCE-2023-0304en.
103. Topshee S, Turner D, Norris M, Harrison M. Exploring the feasibility of an integrated outreach medical clinic within a community-based center for young mothers and their children. J Pediatr Adolesc Gynecol. 2024;37(2 Suppl):262. Presented at: NASPAG 38th Annual Clinical Research Meeting; Orlando, FL, USA.
104. Unger JA, Kinuthia J, Wandika B, Hedstrom A, Wetzler E, Udren JI, et al. The impact of interactive text communication on neonatal mortality in Kenya: a randomized controlled trial. Nat Med. 2025;29:1234-42
105. Werner NE, Morgen M, Kooiman S, Jolliff A, Warner G, Feinstein J, et al. Effectiveness of a Mobile App (Meds@HOME) to Improve Medication Safety for Children With Medical Complexity: Protocol for a Randomized Controlled Trial. JMIR Research Protocols. 2024;13:e60621.
106. Pilarz M, Morales B, Rodriguez V, Jackson K, Pack A, Malakooti M, et al. Family-initiated interpretation: a novel approach to communication in the pediatric ICU. Crit Care Med. 2025;53(Suppl 1):e16. Presented at: 54th Critical Care Congress of the Society of Critical Care Medicine; 2025.
107. Ahmed D, Eapen D, Burmeister B, Parnell P, Brain A, Pal R. Open access (OA) for children in Oxfordshire using local OA-team support: can PDSA interventions improve the experience for parents and staff? Arch Dis Child. 2024;109(Suppl 1):A384-A385. Presented at: Royal College of Paediatrics and Child Health Conference (RCPCH 2024); Birmingham, UK.
108. Flaherty KE, Mahama MN, Klarman MB, Anane-Binfoh NA, Patel MD, Smith NJ, et al. Applying the ADAPT guidance to implement a telemedicine and medication delivery service in a malaria-endemic setting: A prospective cohort study. Tropical Medicine & International Health. 2025;30(3):181-92.
109. Quinonez ZA, Pyke-Grimm KA, Kamra SK, Holmes KE, Char D. Family presence in pediatric cardiac procedural settings: a qualitative study of clinicians, a key stakeholder group. AJOB Empir Bioeth. 2025;16(3):163-71. doi:10.1080/23294515.2025.2474918
110. Madduri GB, Nichols T, Gunturkun F, Johnson D, Lum A, Shaner MA, et al. Improving Family-Centered Rounds With a Nursing Checklist in the Electronic Health Care Record. Hospital Pediatrics. 2024;14(11):919-27.
111. Beard LA, Ceballos KC, Staton KL, Conner KA, Math CM, Moore MK, et al. Reducing NICU Discharge Care–Related Failures by Improving Discharge Safety. Pediatrics. 2025;156(2). doi: 10.1542/peds.2024-066305
